# Proteomics and tracer metabolomics link GAPDH ISGylation to glycolytic control

**DOI:** 10.1186/s13059-026-04034-w

**Published:** 2026-03-11

**Authors:** Denzel Eggermont, Lissa Eggermont, Nagihan Aslantaş, Fabien Thery, Katie Boucher, Antje Beling, Bart Ghesquière, Francis Impens

**Affiliations:** 1 VIB-UGent Center for Medical Biotechnology, VIB, Ghent, Belgium; 2 Department of Biomolecular Medicine, Ghent University, Ghent, Belgium; 3 Present Address: Department of Medical Oncology, Ghent University Hospital, Ghent, Belgium; 4 VIB Proteomics Core, VIB, Ghent, Belgium; 5 Charité, Universitätsmedizin Berlin, Corporate Member of Freie Universität Berlin and Humboldt-Universität Zu Berlin, Institute of Biochemistry, Berlin, Germany; 6 Deutsches Zentrum Für Herz-Kreislauf-Forschung, Partner Site Berlin, Berlin, Germany; 7 Metabolomics Core Facility Leuven, VIB Center for Cancer Biology, VIB, Louvain, Belgium; 8 Laboratory of Applied Mass Spectrometry, Department of Cellular and Molecular Medicine, Katholieke Universiteit Leuven, Louvain, Belgium

**Keywords:** ISG15, Glycolysis, GAPDH, Mass spectrometry, Proteomics, Metabolomics

## Abstract

**Background:**

Ubiquitin-like protein ISG15 (interferon-stimulated gene 15) is implicated in the regulation of central carbon metabolism, but conflicting findings across experimental systems limit mechanistic insight. Here, we apply a multi-omics approach in cells ectopically expressing the ISGylation machinery independent of immune stimuli, to generate a systematic view of ISGylation in metabolic control.

**Results:**

ISGylation preferentially targets metabolic enzymes, with marked enrichment among glycolytic proteins, suppressing the energy-yielding phase of glycolysis. Tracer metabolomics reveals a bottleneck at glyceraldehyde-3-phosphate dehydrogenase (GAPDH), reflected by accumulation of upstream intermediates and depletion of downstream metabolites. This arises from multisite ISGylation of lysines near its catalytic and regulatory regions, which reduces enzymatic activity without disrupting tetramer assembly.

**Conclusions:**

These findings identify GAPDH as a central metabolic checkpoint regulated by ISGylation and uncover a direct post-translational mechanism by which ISG15 controls energy metabolism.

**Supplementary Information:**

The online version contains supplementary material available at 10.1186/s13059-026-04034-w.

## Background

Cellular metabolism is highly adaptable, continuously reprogrammed to meet the energetic and biosynthetic demands of specific physiological states [1]. At the core of this network lies central carbon metabolism, which not only fuels ATP production but also supplies essential precursors for macromolecule synthesis. Glycolysis, a central pathway in this system, converts glucose into pyruvate while generating ATP and reducing equivalents in the form of NADH [2]. Under aerobic conditions, pyruvate is typically funneled into the tricarboxylic acid (TCA) cycle and oxidative phosphorylation (OXPHOS). However, in certain contexts, most notably in cancer, cells shift their metabolism toward aerobic glycolysis, converting glucose to lactate even in the presence of oxygen. This phenomenon, known as the Warburg effect [3], reflects a deliberate reprogramming of energy metabolism that supports rapid proliferation and anabolic growth. A similar metabolic adaptation occurs in immune cells such as macrophages, in which aerobic glycolysis is upregulated during M1 polarization to fuel their pro-inflammatory activity [4].

The metabolic reprogramming of immune cells during activation is a central focus in the emerging field of immunometabolism [5]. Various signals, including cytokines and damage-associated or pathogen-associated molecular patterns (DAMPs and PAMPs), drive important metabolic shifts through activation of innate immune pathways. Type I interferon (IFN) signaling plays a crucial role in this process, coordinating a robust antimicrobial response by inducing the expression of interferon-stimulated genes (ISGs) and simultaneously influencing key metabolic pathways, such as glycolysis, the TCA cycle and OXPHOS [6, 7]. These metabolic adaptations are vital for effective immune responses but can also be manipulated by pathogens, which often hijack host metabolic processes to enhance their own replication and survival [8, 9]. Among the ISGs, the ubiquitin-like protein ISG15 has recently been linked to the regulation of cellular metabolism during immune activation, potentially counteracting these pathogenic strategies [10].

ISG15 is expressed as a 17 kilodalton (kDa) precursor that is rapidly cleaved into a 15 kDa mature form [11]. Processing of ISG15 reveals a C-terminal LRLRGG motif that allows conjugation onto target proteins. The covalent attachment of ISG15 proceeds through multiple steps in a process called ISGylation. An E1-activating enzyme (UBA7) binds and activates ISG15 [12]. Activated ISG15 is transferred to an E2-conjugating enzyme (UBE2L6) [13] and in the final step, an E3 ligase (human HERC5/mouse HERC6, TRIM25, ARIH1) catalyzes the conjugation of ISG15 onto the ε-amine of a target lysine residue [14–17]. In addition, ISGylation can be reversed through the action of deconjugating proteases such as USP16 and USP18 [18, 19].

ISG15 has been primarily studied in the context of viral infection where ISGylation of host and viral proteins was shown to protect the cell against infection [20]. Numerous in vivo studies in mice lacking ISG15 have demonstrated increased susceptibility to a wide range of viruses, including murine gammaherpesvirus, influenza A virus, influenza B virus, Sindbis virus, vaccinia virus, coxsackievirus B3, herpes simplex virus 1, Chikungunya virus and murine norovirus [21–26]. This broad antiviral function of ISG15 may be linked to HERC5’s association with polysomes, which primes newly translated proteins for ISGylation [27]. Consequently, viral proteins are primary targets of the ISG15 conjugation system, leading to a loss of their function upon modification. In particular, ISGylation of viral proteins can disrupt their interaction with host pathways required for replication, limit their ability to disrupt the innate immune response or interfere with virion self-assembly [20].

This mechanism, however, does not fully account for the antibacterial role of ISG15. Unlike viruses, intracellular bacteria rely on their own translation machinery and are functionally more independent within host cells, making their proteins less susceptible to the effects of ISGylation. In line with this, only a few bacterial proteins have been found to be ISGylated during infection [28]. Despite this, ISG15 has demonstrated effectiveness against intracellular bacterial pathogens, including *Mycobacterium tuberculosis* and *Listeria monocytogenes* [29, 30]. This, coupled with the fact that hundreds of host proteins are modified by ISG15 [31], indicates that ISGylation of host proteins adds additional layers to ISG15’s role in antimicrobial defense. Supporting this, several key immune effectors such as cGAS [32, 33], STING [34], and MDA5 [35] have recently been identified as ISG15 targets that are notably activated upon ISGylation, thereby enhancing their antimicrobial activity.

Beyond innate immune proteins, also metabolic proteins are consistently identified as major targets of ISGylation in proteomics screens [31]. In the livers of mice infected with *Listeria monocytogenes*, a high number of metabolic enzymes were found to be modified by ISG15 with modification sites close to the active site, potentially reducing their activity [28]. Likewise, HERC5-dependent ISG15 sites in human cells were significantly enriched on glycolytic and TCA cycle proteins in line with their high translation efficiency and the co-translational model of ISGylation [36]. Similar findings were observed in porcine alveolar macrophages, where metabolic proteins were prominently represented among ISGylated targets [37]. Finally, the regulatory significance of these modifications has been highlighted by studies showing that both host and viral ISG15 proteases can remove ISG15 from these substrates [19, 38].

Despite these insights on the protein level, understanding the precise metabolic effects of ISG15 remains challenging due to conflicting findings across various cell types and conditions. Focusing on glycolysis, IFN-stimulated human macrophages and fibroblasts exhibit increased glycolysis in the absence of ISG15 [39]. In contrast, IFN-treated *Isg15*^*−/−*^ bone marrow-derived macrophages show no significant changes in glycolysis [40], while human *ISG15*^*−/−*^ pancreatic cancer stem cells demonstrate a substantial decrease in glycolytic activity [41]. Meanwhile, studies in *Isg15*^*−/−*^ mice consistently identify ISG15 as a negative regulator of glycolysis, where it inhibits glycolysis by modifying key enzymes, offering protection against coxsackievirus infection and high-fat diet-induced obesity [10, 42].

These apparent discrepancies may arise from the use of different immune stimulants to induce ISGylation, as each stimulant impacts metabolism through various pathways, with ISGylation being just one factor. To isolate the intrinsic effects of ISG15 on metabolism, we employed a multi-omics approach using human HeLa cells that express the ISGylation machinery independent of immune stimuli. We show that ISGylation predominantly targets metabolic proteins, with multiple modification sites identified on key enzymes of the glycolytic pathway. This modification is associated with a marked suppression of glycolysis, particularly affecting the reaction catalyzed by glyceraldehyde-3-phosphate dehydrogenase (GAPDH), further supported by in vitro enzyme assays and structural insights. Together, we propose GAPDH as an important metabolic checkpoint in the ISGylation-dependent regulation of glycolysis.

## Results

### Proteomics validation of a cellular ISGylation model

To investigate the role of ISGylation in metabolism, we set up a cellular system that induces ISGylated proteins both within and independent of an IFN response. HeLa cells were either treated with IFN or transiently transfected to overexpress the ISG15 conjugation machinery (E1, E2, E3, and ISG15). In both scenarios, we observed a substantial increase in free ISG15 levels alongside an accumulation of ISGylated proteins compared to mock-transfected control cells (Additional file 1: Fig. S1). To validate our system, we conducted a proteome-wide analysis of ISG15 modification sites and compared our data with previously reported datasets to evaluate the overlap and confirm the robustness of our approach. We relied on a commercially available kit for mapping ubiquitin sites that was previously used for ISG15 [28]. During trypsin digestion, ISG15 conjugates are cleaved, leaving a diglycine remnant (GG) on the modified lysine residues. By enriching for diglycine-modified peptides with anti-K-ε-GG antibodies, the modified peptides and their exact site of modification can be identified by mass spectrometry (MS)-based proteomics. As ubiquitin and NEDD8 produce the same diglycine adducts after trypsin digestion, we included *ISG15*^*−/−*^ controls to ensure the specificity of ISG15 site assignment, as established in prior ISGylome studies [10, 28, 38].

**Fig. 1:**
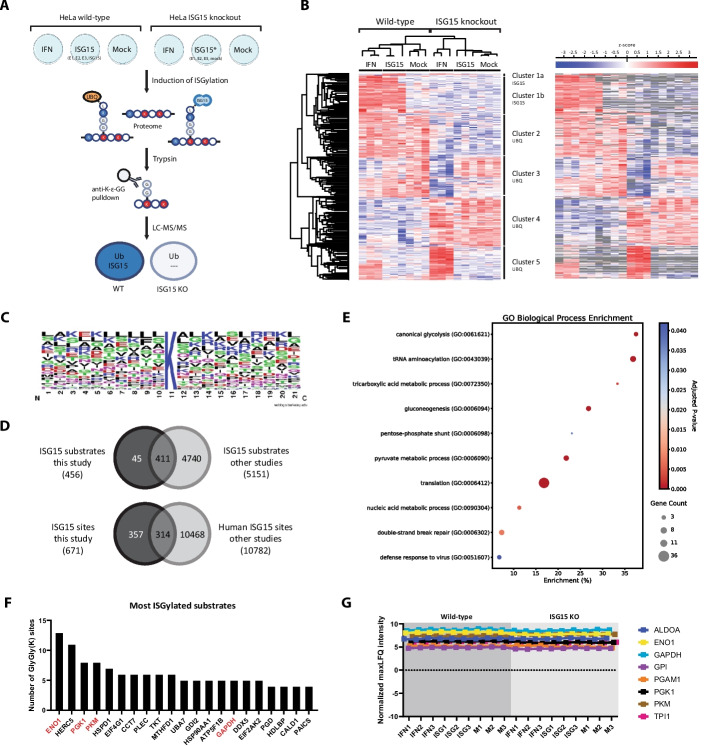
Identification of the ISGylome in HeLa cells upon IFN stimulation and ectopic expression of the ISGylation machinery. **A** Schematic overview of the experimental design for ISG15 site mapping by mass spectrometry. Wild-type and *ISG15* knockout (KO) HeLa cells were either mock-transfected (Mock), IFN-stimulated (IFN), or transfected with the ISG15 conjugation machinery (ISG15). In *ISG15* KO cells, the conjugation machinery was expressed without ISG15. After treatment, cells were lysed and proteins digested with trypsin, leaving a diglycine (GG) remnant on ISG15-modified lysines. GG-modified peptides were enriched using anti-K-ε-GG antibodies and analyzed by LC–MS/MS. ISG15 sites were identified by comparing peptide profiles from wild-type and *ISG15* KO samples. Figure created in Biorender: https://BioRender.com/cd1fmsv. **B** Left, heatmap showing significantly regulated GlyGly(K) sites after unsupervised hierarchical clustering (n = 3 independent biological repeats). Different genotypes (wild-type or *ISG15* KO) and treatments (IFN, ISG15, or Mock) are indicated. The colors represent upregulated (red) or downregulated (blue) sites. Right, the same heatmap is shown with the originally missing values depicted in gray. Five major clusters can be observed that either contain ISG15 sites (cluster 1a/1b), or ubiquitin sites (cluster 2–5). **C** Sequence logo drawn based on sequence alignment of the ISG15 sites and their + 7 and −7 flanking residues. **D** Venn diagram showing the overlap between ISG15 substrates identified in this study and those reported across species, and between ISG15 modification sites identified here and in previous human studies. **E** Gene ontology analysis of biological processes enriched among ISG15 substrates, visualized in a bubble plot. Enrichment of ISG15 substrates in each functional category was determined by Fisher's exact test (*P* < 0.05). The vertical axis represents biological process categories, and the horizontal axis shows gene enrichment (%). Bubble size corresponds to the number of ISG15 substrates in each category, while bubble color indicates the *p*-value. **F** Bar chart showing the most modified ISG15 substrates in this study (substrates with ≥ 4 sites). **G** Profile plot showing maxLFQ intensity for glycolytic enzymes targeted by ISG15 across genotype and treatments, based on shotgun proteomics

We mapped ISG15 sites across three conditions: mock-transfected control cells (mock), IFN-induced cells (IFN) and cells transfected with the ISGylation machinery (ISG15) (Fig. 1A). Prior to proteomics analysis, we checked the quality of our samples by Western blot. As anticipated for the wild-type (WT) cell line, no ISGylation was detected in the mock condition, while clear induction of ISGylation was seen in the IFN and ISG15 conditions (Additional file 1: Fig. S2). In contrast, no ISGylation was detected in the *ISG15*^*−/−*^ cell line upon IFN treatment or transfection of the conjugation machinery without ISG15 (E1, E2 and E3). Next, we used liquid chromatography-tandem mass spectrometry (LC–MS/MS) to identify modification sites by enriching for peptides that carry a diglycine remnant after digestion with trypsin. Following statistical analysis and unsupervised hierarchical clustering, significantly regulated sites grouped into five clusters (Fig. 1B) (Additional file 2: Table S1). Replicate cell cultures also clustered together by genotype and condition, indicating the high reproducibility of our approach.

In total, we identified 671 ISG15 sites on 456 proteins present in cluster 1 (Fig. 1B). These sites were induced upon IFN treatment and overexpression of the ISGylation machinery (Fig. 1B, cluster 1a and 1b), but they were generally absent in *ISG15*^*−/−*^ control cells as indicated in gray (Fig. 1B, right panel), and are therefore considered *bona fide* ISG15 sites. The majority of the ISG15 sites overlapped between the IFN and ISG15 conditions, indicating that both treatments produced a similar ISGylation pattern as reported before [36] (Fig. 1B, cluster 1b). In addition, we found 20 sites specifically upregulated in the overexpression condition (Fig. 1B, cluster 1a). Many of these sites occurred on UBA7 and HERC5, the ISG15 E1 and E3 enzyme, respectively, consistent with previous reports that overexpressed proteins are primary targets of ISGylation according to the co-translational model of ISGylation (Additional file 1: Fig. S3) [27]. We also analyzed the primary amino acid sequences of identified ISG15 sites and found no clear consensus motif for ISG15 modification, in line with previous reports [10, 28, 36] (Fig. 1C). Finally, we compared our identified ISG15 substrates and sites with existing datasets from previous studies [10, 31, 36, 38, 42]. We observed a 90% overlap at the substrate level and a 47% overlap at the site level, underscoring the high quality of our dataset (Fig. 1D).

In addition to ISG15 sites, we identified 2,672 significantly regulated ubiquitination sites across 1,378 proteins localized in clusters 2, 3, 4, and 5 (Fig. 1B). Among these, 790 sites were significantly downregulated in WT cells regardless of treatment, likely reflecting genotype-dependent differences (Fig. 1B, cluster 4). We further identified 684 ubiquitin sites upregulated in the WT cells (Fig. 1B, cluster 2). These sites could also be marked as ISG15 sites present under basal conditions, but are more likely to be ubiquitin sites differently expressed between WT and *ISG15*^*−/−*^ cells as these sites were not completely absent in the *ISG15*^*−/−*^ cells. In addition, we identified 558 ubiquitin sites that were upregulated upon IFN treatment with a more pronounced increase in *ISG15*^*−/−*^ cells (Fig. 1B, cluster 5). Consistently, 640 ubiquitin sites were specifically downregulated in *ISG15*^*−/−*^ cells upon IFN treatment, indicating an altered IFN response in the absence of ISG15, consistent with previous reports [38, 43] (Fig. 1B, cluster 3).

### ISG15 targets glycolytic enzymes

To gain insights into the functional implications of ISGylation, we performed a gene ontology analysis of ISGylated proteins using PANTHER via the Gene Ontology Resource [44–46], focusing on biological processes (Fig. 1E). This analysis revealed a significant enrichment in several metabolic pathways, including canonical glycolysis, gluconeogenesis, the TCA cycle, the pentose-phosphate shunt, as well as pyruvate and nucleic acid metabolism. Consistent with these findings, nearly all glycolytic enzymes were found to be ISGylated, with four ranking among the top ISGylated proteins in our dataset (Fig. 1F). This extensive ISGylation of glycolytic enzymes has been previously reported in mice and is associated with decreased glycolytic activity [10, 42]. Additionally, we observed an enrichment in biological processes such as tRNA aminoacylation and translation, reflecting the association of HERC5 with polysomes and the co-translational model of ISGylation [47]. We also noted enrichment in proteins involved in double-strand break repair and defense response to virus, consistent with ISG15’s role in DNA replication stress and antiviral defense [20, 48]. Overall, the marked enrichment of metabolic functions and processes among ISGylated proteins, including key glycolytic enzymes, indicates a significant role of ISGylation in cellular metabolism.

To confirm that proteome alterations in the *ISG15*^*−/−*^ cells did not lead to false positive identifications of ISG15 sites on these glycolytic enzymes (for example, by downregulation of ubiquitinated proteins in these cells), we performed a standard shotgun proteomics analysis of the input samples to highlight differentially regulated proteins between WT and *ISG15*^*−/−*^ cells (Additional file 1: Fig. S4) (Additional file 2: Table S2). Comparison of protein intensities between WT and *ISG15*^*−/−*^ samples revealed 246 significantly regulated proteins, of which 120 were downregulated in *ISG15*^*−/−*^ cells (Additional file 2: Table S3). ISG15 showed the greatest downregulation in *ISG15*^*−/−*^ cells, consistent with its absence in these samples. Of the identified ISG15 sites, only 17 were found on significantly regulated proteins, precluding any major effect of genotype on ISG15 site identification (Additional file 2: Table S3). All other identified ISG15 sites (clusters 1a and 1b), including those on glycolytic enzymes, showed no significant differences in protein intensities across conditions (Fig. 1G). Altogether, these data confirm that the detected ISG15 sites correspond to *bona fide* ISGylation sites, not resulting from protein level changes across conditions.

### ISG15 is a negative regulator of glycolysis at energy-producing steps

**Fig. 2:**
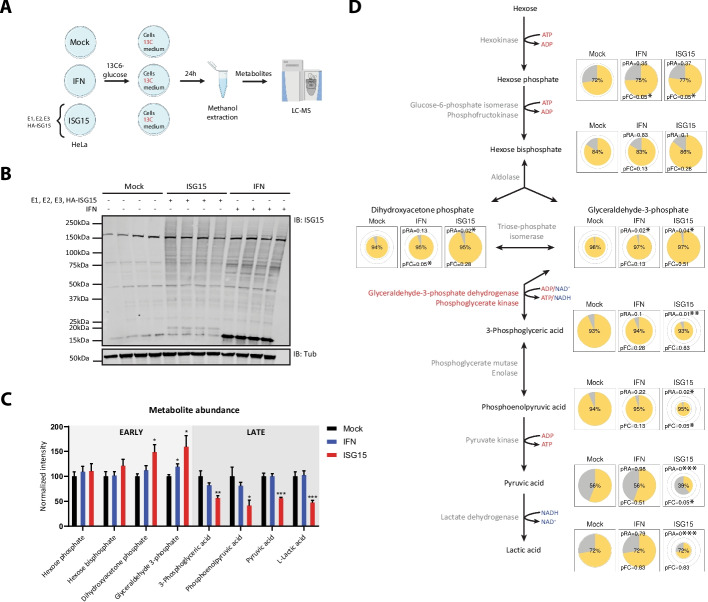
ISGylation targets the conversion of glyceraldehyde-3-phosphate to 3-phosphoglyceric acid to suppress glycolysis. **A** Schematic overview of the tracer metabolomics experiment. Mock-transfected control cells (Mock), IFN-treated cells (IFN), and cells transfected with the ISGylation machinery (ISG15) were incubated with ^13^C_6_-glucose for 24 h, followed by metabolite extraction and LC–MS analysis. Figure created in Biorender: https://BioRender.com/a5gbctg. **B** Quality control of ISGylation induction in cells used for metabolomics. ISG15 conjugates were detected by immunoblotting with anti-ISG15 antibody (IB:ISG15). α-Tubulin was used as a loading control (IB:Tub). **C** Relative abundances of glycolytic intermediates across Mock, IFN, and ISG15 conditions. The abundance of each metabolite is shown relative to the mock condition. **D** TraVis Pie visualizations of the metabolomics data mapped onto the glycolytic pathway, summarizing metabolite abundance (same as in C) and ^13^C_6_-glucose-derived labeling across conditions (Mock, IFN and ISG15). Pie radii indicate relative metabolite abundance, while the yellow segment and the central percentage denote the fractional contribution from labeled glucose. The remaining segment (gray) represents the fractional contribution from unlabeled sources. Statistical significance for relative abundance (pRA) and fractional contribution (pFC) is shown relative to mock. **C**,** D** Comparisons were made using two-tailed Student’s t-tests. Data are presented as mean ± SEM (*n* = 3 independent biological repeats). * *p* < 0.05, ** *p* < 0.01, *** *p* < 0.001

Given the enrichment of metabolic proteins modified by ISG15, we next investigated whether these modifications affected metabolite levels in central carbon metabolism. For this, we set up a tracer metabolomics experiment in our cellular ISGylation model consisting of mock-transfected control cells (mock), IFN-induced cells (IFN) and cells transfected with the ISG15 conjugation machinery (ISG15). Following induction of ISGylation, the medium of the cells was replaced with medium containing isotopically labeled ^13^C_6_-glucose to monitor incorporation of the isotopic tag into different biochemical pathways (Fig. 2A). This approach enabled us to examine both metabolite abundances and the activity of key metabolic routes [49]. Specifically, we traced metabolites from two major pathways, including glycolysis and the TCA cycle, which have previously been implicated in ISGylation-induced effects on cellular metabolism [10, 40, 42].

Prior to metabolomics analysis, we verified our setup by Western blot. As expected, no ISGylation could be detected in the mock condition, while clear induction of ISGylation was seen in the IFN and ISG15 conditions (Fig. 2B). Metabolites from these samples were subsequently analyzed using high-throughput liquid chromatography-mass spectrometry (LC–MS) analysis (Additional file 2: Table S4). Principal component analysis of metabolite abundances revealed clear separation among the three conditions and showed that replicate samples within each condition clustered together, indicating high quality of the dataset (Additional file 1: Fig. S5). Next, we used TraVis Pies for an integrated view of the average relative abundance (radius or RA) and fractional contribution (FC) of the ^13^C_6_-glucose tracer into each metabolite of the glycolytic pathway and TCA cycle [49]. At the start of glycolysis, no significant differences in hexose phosphate and hexose bisphosphate among the three conditions were observed (Fig. 2C, D). These metabolites are collectively referred to as hexose derivatives here, as isomeric species like glucose-1-phosphate and glucose-6-phosphate cannot be reliably distinguished by conventional LC–MS [50]. By contrast, dihydroxyacetone phosphate and glyceraldehyde-3-phosphate showed a statistically significant accumulation in the ISG15 condition compared to the mock control (Fig. 2C, D). The same effect was present in the IFN condition, though less pronounced. This strong accumulation coincided with reduced abundances of lower glycolysis intermediates such as 3-phosphoglyceric acid, phosphoenolpyruvic acid and pyruvic acid in the ISG15 condition compared to the mock control, indicating an inhibitory effect of ISGylation on the reactions catalyzed by GAPDH and phosphoglycerate kinase 1 (PGK1) (Fig. 2C, D). A similar trend occurred under IFN conditions but without significant differences in phosphoenolpyruvic acid and pyruvic acid (Fig. 2C, D). Interestingly, in the ISG15 condition, the reduced abundance of pyruvic acid was partly rescued by increased uptake or conversion of pyruvate from alternative pathways as indicated by a higher fraction of unlabeled pyruvate compared to the mock control (respectively 61% versus 44%, Fig. 2D). Collectively, our data indicate that ISGylation disrupts glycolysis by interfering with the conversion of glyceraldehyde-3-phosphate to 3-phosphoglyceric acid, catalyzed by the glycolytic enzymes GAPDH and PGK1.

Within the TCA cycle, we observed reduced abundances of oxoglutaric acid and oxaloacetic acid in the ISG15 condition compared to the control (Additional file 1: Fig. S6). Additionally, succinic acid levels were lower in both the ISG15 and IFN conditions. However, due to the minimal tracer incorporation into the TCA cycle intermediates (Additional file 1: Fig. S6), we chose not to pursue further analysis of TCA cycle involvement. This limited incorporation of glucose carbons into the Krebs cycle may be linked to the Warburg effect, in which cancer cells like HeLa cells preferentially convert glucose to lactate even in the presence of oxygen [3]. Notably, in the ISG15 condition, lactate levels were significantly reduced compared to the mock control, consistent with the observed decrease in other lower glycolysis intermediates (Fig. 2D). Collectively, these data offer the first systematic insight into how ISGylation affects central carbon metabolism, with its primary effect observed in the energy-producing steps of the glycolytic pathway.

### Impact of ISGylation on GAPDH and PGK1 activity

**Fig. 3:**
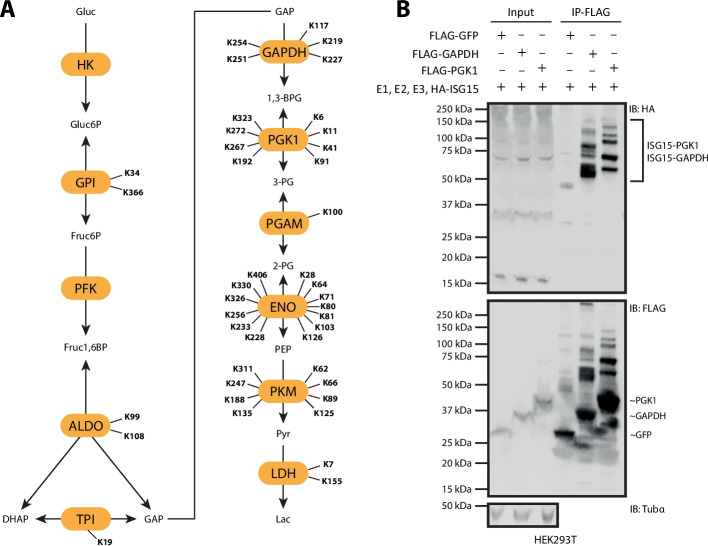
ISG15 modifies glycolytic enzymes GAPDH and PGK1. **A** Schematic overview of glycolysis highlighting enzymes with ISG15 modification sites identified in our ISGylomics screen. **B** Validation of ISG15 modification of glyceraldehyde-3-phosphate dehydrogenase (GAPDH) and phosphoglycerate kinase (PGK1). HEK293T cells were transfected with the ISGylation machinery (E1, E2, E3, and HA-ISG15) and FLAG-tagged GFP, GAPDH, or PGK1, followed by FLAG immunoprecipitation (IP) and Western blot analysis. Blots were probed with anti-HA to confirm conjugation (IB:HA), anti-FLAG to detect GFP, GAPDH or PGK1 (IB:FLAG), and anti-α-tubulin as a loading control (IB:Tubα). Images are representative of three independent biological repeats (*n* = 3)

The metabolomics analysis revealed that ISGylation inhibits glycolysis at the steps catalyzed by GAPDH and PGK1. Given that both enzymes were identified as hits in our proteomics screen (Fig. 3A), we hypothesized that their ISGylation drives this metabolic effect. To test this, we first confirmed whether GAPDH and PGK1 are modified by ISG15. We performed pull-down assays with FLAG-GAPDH or FLAG-PGK1 from lysates of HEK293T cells expressing the ISG15 conjugation machinery (E1, E2, E3 and ISG15). Immunoblot analysis showed a distinct ladder pattern for GAPDH and PGK1, indicative of single or multiple ISG15 modifications, which was absent in the FLAG-GFP pull-down control (Fig. 3B). This result aligns with our proteomics analysis, which identified multiple ISG15 modification sites on both GAPDH and PGK1, and is consistent with previous reports of ISGylation on GAPDH (Fig. 3A) [10, 51].

To further validate the modification sites, we performed affinity-purification mass spectrometry (AP-MS) on GAPDH and PGK1 immunoprecipitates and mapped GlyGly(K) remnants indicative of ISG15 conjugation. A conjugation-defective HA-ISG15-AA mutant was included to distinguish ISG15 from ubiquitin-derived GlyGly(K) sites. Using this approach, we recovered five previously reported ISG15 sites on GAPDH [31], including K219 and K227, which were also identified in our ISGylomics dataset (Additional file 1: Fig. S7). Similarly, six established sites were detected on PGK1 [31], four of which (K41, K91, K267, K323) overlapped with our initial screen (Additional file 1: Fig. S7). The high degree of concordance between the AP-MS data and our global profiling screen confirms and extends our site-mapping analysis, supporting the direct ISGylation of GAPDH and PGK1.

**Fig. 4:**
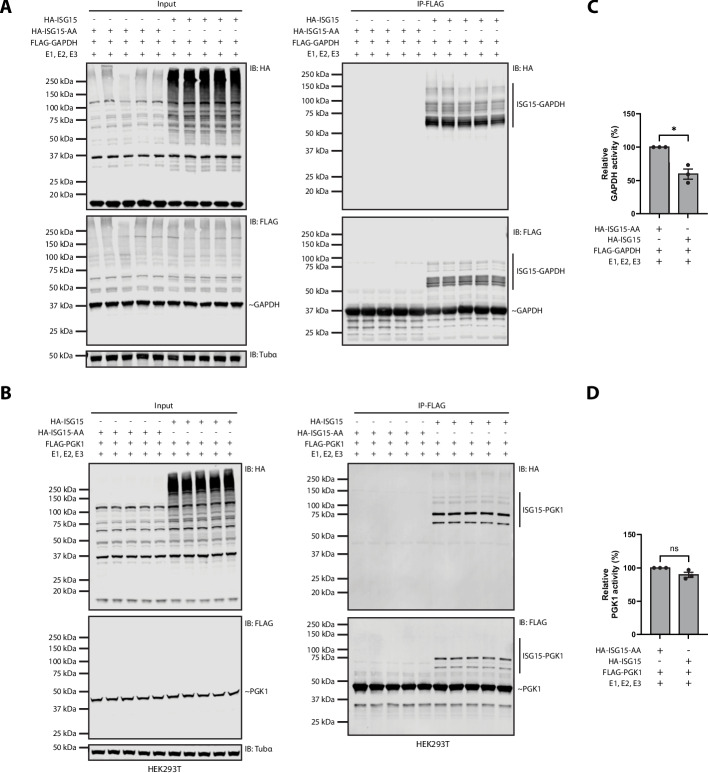
ISGylation reduces GAPDH activity, but not PGK1 activity in vitro. **A**, **B** FLAG-tagged GAPDH (**A**) or FLAG-tagged PGK1 (**B**) was immunoprecipitated from HEK293T cell lysates co-expressing either HA-ISG15 or the conjugation-defective mutant HA-ISG15-AA, along with the ISG15 conjugation enzymes (E1, E2, and E3). Western blot analysis confirmed successful ISGylation and target pulldown. Representative blots from three independent biological repeats are shown. Membranes were probed with anti-FLAG to detect the target proteins (IB:FLAG), anti-HA to confirm conjugation (IB:HA), and anti-α-tubulin as a loading control (IB:Tubα). ISG15-modified forms are indicated, while unmodified proteins are marked with a tilde (~). **C**, **D** Enzymatic activity assays were performed on immunoprecipitated GAPDH (**C**) or PGK1 (**D**). Activities were normalized to immunoprecipitated protein levels and expressed relative to baseline across three independent biological repeats (*n* = 3), each with five technical replicates per condition. Statistical comparisons were made using two-tailed t-tests. Data are represented as mean ± SEM; * *p* < 0.05; ns, not significant

With these targets validated, we next investigated whether ISGylation affects their catalytic activity. We immunoprecipitated FLAG-GAPDH or FLAG-PGK1 from lysates of HEK293T cells co-expressing HA-ISG15 or the conjugation-defective mutant HA-ISG15-AA, along with the conjugation enzymes. Immunoblot analysis confirmed the presence of ISGylated species at higher molecular weights under wild-type conjugation conditions, whereas only unmodified protein was recovered in the ISG15-AA control (Fig. 4A, B). The enriched enzymes were subsequently incubated with their respective substrates, and catalytic activity was assessed using a colorimetric assay. Following ISG15 modification of GAPDH, its catalytic activity was significantly reduced by up to 50% compared to GAPDH enriched from cells lacking ISGylation (Fig. 4C). In contrast, PGK1 activity was unaffected by ISGylation under the same conditions (Fig. 4D). Together, these findings indicate that ISGylation suppresses glycolysis primarily by modifying GAPDH and reducing its enzymatic activity.

### ISGylation inhibits GAPDH activity independent of oligomerization

**Fig. 5:**
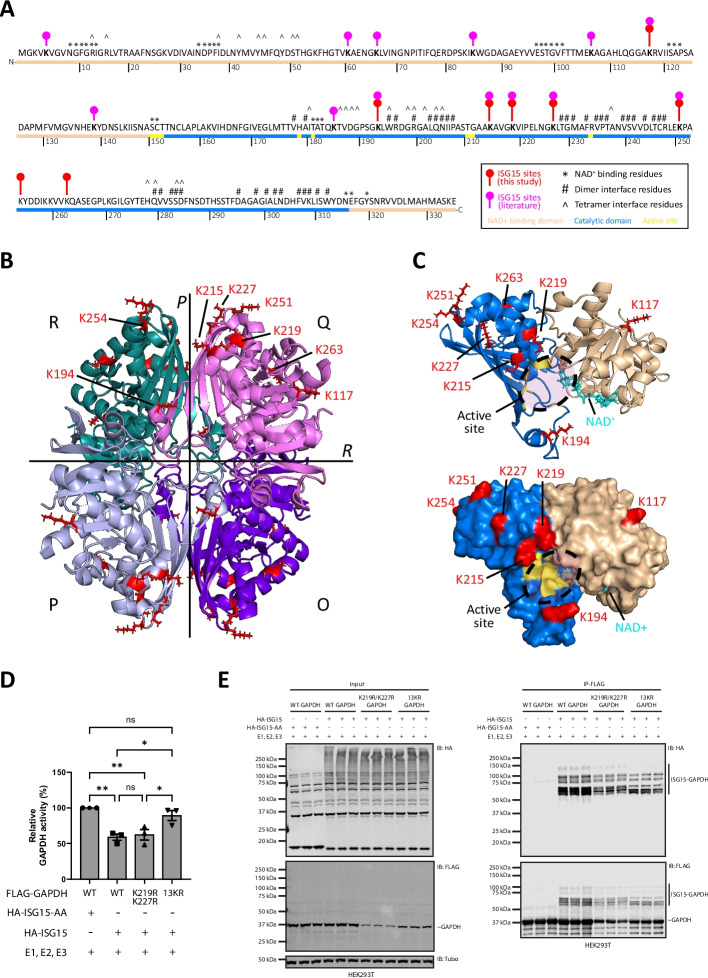
Structural insights into ISG15-mediated inhibition of GAPDH. **A** ISG15 modification sites identified in this study (red) and recurrently detected in other studies (magenta) are mapped onto the amino acid sequence of wild-type human GAPDH (Additional file 2: Table S5) [10, 28, 36–38, 54]. The domain organization of GAPDH is shown, with the NAD⁺-binding domain (residues 1–150 and 317–335) colored beige, and the catalytic domain (residues 151–316) colored blue. The active site, indicated in yellow, contains the catalytic residues S151, C152, and H178, as well as residues involved in substrate binding (T153, T182, T211, G212, and R234) [55]. Residues involved in the dimer interface (#), tetramer interface (^), and NAD⁺ binding (*) are annotated above the sequence. **B** GAPDH consists of four identical subunits (O, P, Q, R), forming a tetrameric quaternary structure. The subunits are related by three twofold symmetry axes (*P, Q, R*; *Q* is not shown). The dimer interface, located along the P axis (between monomers R and Q or between P and O), and the tetramer interface, located along the R axis (between dimers R/Q and P/O), are indicated. ISG15 modification sites identified in this study are highlighted in red (stick representation) on the GAPDH crystal structure (PDB 4WNC, cartoon). **C** A single GAPDH subunit is shown with the identified ISG15 modification sites highlighted in red (stick representation), displayed in both cartoon (top) and surface (bottom) views (PDB 4WNC). The NAD⁺-binding domain is colored beige, the catalytic domain blue, and the active site yellow, marked by a circle at the interface of the two domains. An NAD⁺ molecule bound to the subunit is shown in cyan (stick representation). Models were created using PyMOL Molecular Graphics System v3.1.3 (Schrödinger, LLC). **D** FLAG-tagged wild-type or lysine-to-arginine (KR) mutant GAPDH was immunoprecipitated from HEK293T cells co-expressing either HA-ISG15 or the conjugation-defective mutant HA-ISG15-AA, along with the ISG15 conjugation enzymes (E1, E2, E3). Immunoprecipitated material was used for enzymatic activity assays, with activities normalized to immunoprecipitated protein levels and expressed relative to baseline across three independent biological repeats (*n* = 3), each with three technical replicates per condition. Statistical significance was assessed by one-way ANOVA with Tukey’s post-hoc test. Data are presented as mean ± SEM. * *p* < 0.05, ** *p* < 0.01; ns, not significant. **E** Representative Western blot of the samples in (D) confirming successful ISGylation and pulldown. Membranes were probed with anti-FLAG to detect GAPDH (IB:FLAG), anti-HA to confirm ISG15 conjugation (IB:HA), and anti-α-tubulin as a loading control (IB:Tubα). ISG15-modified forms are indicated, whereas unmodified proteins are marked with a tilde (~)

To investigate the mechanism underlying ISG15-dependent inhibition of GAPDH activity, we mapped our identified ISG15 modification sites onto the crystal structure of GAPDH [52]. GAPDH is a 335-amino-acid enzyme consisting of an NAD⁺-binding domain (residues 1–150, 317–335) and a catalytic domain (residues 151–316), which contains the active site cysteine (Fig. 5A). The enzyme predominantly exists as a homotetramer composed of two dimers, with the four subunits arranged along three two-fold symmetry axes (P, Q and R) [53]. The P axis defines the dimerization interface (e.g., monomers P-O or Q-R), while the R axis defines the interface between the two dimers that form the tetramer (Fig. 5B).

Several ISG15 sites, including K215, K227 and K254, are located near the dimerization interface (Fig. 5B). These residues cluster along the P axis, which mediates monomer–monomer contacts required for dimer formation and, subsequently, tetramer assembly [56]. To test whether ISGylation inhibits GAPDH by destabilizing higher-order oligomers, we first assessed the oligomeric state of endogenous GAPDH in HeLa cells. Crosslinking revealed similar levels of dimers and tetramers regardless of ISGylation (Additional file 1: Fig. S8A, B). To more sensitively detect potential differences, we repeated the analysis in HEK293T cells expressing FLAG-GAPDH together with the ISGylation machinery, allowing isolation of modified and unmodified GAPDH by immunoprecipitation prior to crosslinking. Again, we observed no detectable differences in dimer and tetramer formation (Additional file 1: Fig. S8C). Instead, ISGylated GAPDH migrated as a high-molecular-weight species consistent with intact tetramers bearing ISG15. These results indicate that ISGylation does not inhibit GAPDH by disrupting dimer or tetramer assembly. The dimer interface yet also mediates cooperative NAD⁺ binding, with conformational changes in one subunit transmitted to the other [57]. ISGylation at interface-proximal lysines could therefore perturb allosteric coupling even without altering oligomer stoichiometry. In addition, several ISG15 sites identified in our dataset and previous proteomics studies map near the catalytic cysteine and NAD⁺-binding pocket, positioning ISG15 to sterically hinder cofactor or substrate access or altering conformational transitions required for catalysis [58]. To test the functional contribution of these sites, we generated lysine-to-arginine (KR) site mutants to prevent ISG15 conjugation of GAPDH. We first mutated K219 and K227, two residues consistently identified in both our ISGylomics and AP-MS datasets and positioned adjacent to the active site cleft (Fig. 5C). The K219R/K227R mutant displayed reduced enzymatic activity upon ISGylation, similar to wild-type GAPDH, with only a small, non-significant rescue in activity observed (Fig. 5D). This mutant, however, remained robustly ISGylated with multiple high–molecular-weight species, indicating compensatory modification of nearby lysines, suggesting that blocking individual sites alone is insufficient to prevent functional inhibition (Fig. 5E).

Accordingly, we constructed a broader 13-lysine-to-arginine mutant (13KR), encompassing clustered sites near the active site and additional lysines across both GAPDH domains that were repeatedly identified as ISG15 sites across previous studies (Fig. 5A, indicated in magenta) (Additional file 2: Table S5) [10, 28, 36–38, 54]. Notably, 13KR GAPDH showed diminished ISGylation, appearing as discrete modified species, in contrast to the heterogeneous smear of wild-type GAPDH, which reflects ISG15 attachment at multiple alternative lysines in varying combinations. (Fig. 5E). Moreover, this decrease in ISGylation coincided with a substantial restoration of catalytic activity, approaching the baseline levels of unmodified GAPDH (Fig. 5D).

Together, these results directly link GAPDH inhibition to ISG15 modification at a defined set of lysine residues. Our mutagenesis data, together with structural mapping, support a model in which ISG15 suppresses GAPDH activity primarily by interfering with its catalytic function. This occurs through steric obstruction or disruption of conformational dynamics near the active site and allosteric regions, without disrupting oligomeric assembly. The functional specificity of ISG15 modification is further illustrated by PGK1, which shows no loss of activity, consistent with all ISG15 sites mapped in this study residing in regions distant from the active site [59] (Additional file 1: Fig. S9).

## Discussion

ISG15 has long been recognized for its role in immune response pathways, but its contribution to metabolic regulation remains less well understood [20, 31, 38]. Our study identifies GAPDH as a principal metabolic checkpoint modulated by ISGylation to regulate glycolysis. We show that ISG15 modification of GAPDH results in accumulation of upstream substrates and depletion of downstream glycolytic intermediates, consistent with reduced catalytic activity. This effect is mediated through coordinated ISGylation at lysines proximal to the catalytic and regulatory regions, without perturbing tetramer assembly. These results link ISGylation to metabolic control, revealing a direct post-translational mechanism that suppresses glycolysis and highlighting ISG15’s broader role in maintaining metabolic homeostasis. These findings suggest that ISG15 is not only an immune modulator but also has a function in cellular energy regulation.

To investigate the role of ISG15 in metabolism, we established a cellular model by transfecting HeLa cells with the ISGylation machinery, allowing us to study ISG15 modification independent of immune stimuli. In parallel, we compared this model to HeLa cells treated with IFN, a stimulus commonly used to induce an ISGylation response within the context of immune activation [27]. To validate our approach, we employed proteomics with a GlyGly(K)-peptide enrichment strategy to map ISG15 modification sites [28]. In total, we identified 671 ISG15 modification sites on 456 proteins, representing a substantial expansion of the ISGylome in HeLa cells. The dataset shows strong agreement with previous studies, including 47% overlap at the site level and 91% at the protein level [10, 31, 36, 38, 42], and extends the first HeLa ISGylome characterized under SARS-CoV-2 PLpro treatment [38]. Notably, ISGylation profiles induced by IFN treatment or by ectopic expression of the ISGylation machinery were highly concordant, highlighting the robustness of our system and supporting previous reports that reconstituted ISGylation without IFN recapitulates physiologic substrates [36]. Gene ontology analysis further revealed significant enrichment of metabolic processes, with glycolysis as the most prominent pathway. Notably, ISG15 modifications were detected on nearly all glycolytic enzymes, many carrying multiple modification sites. Several of these enzymes ranked among the most ISGylated proteins in our dataset. Among them, GAPDH stood out as a major ISG15 target, consistent with its identification as one of the top ISGylated proteins across previous human, mouse, and pig ISGylome studies [31].

To determine whether widespread ISGylation of glycolytic enzymes produces functional metabolic effects, we performed targeted ^13^C_6_-glucose tracer metabolomics in cells with active ISGylation. As the first tracer-based metabolomics study in the ISGylation field to simultaneously capture all major glycolytic intermediates, our approach offers a more complete picture of the cellular metabolic state than earlier efforts that focused on individual metabolites or overall flux [10, 39–42]. This analysis revealed that ISGylation generally suppresses glycolysis, as evidenced by reduced levels of late-stage intermediates and decreased ^13^C_6_-glucose incorporation into pyruvate. These observations are consistent with studies in ISG15-deficient systems, which exhibit elevated glycolytic rates under both basal and IFN-stimulated conditions, suggesting that ISG15 acts as a molecular brake on glycolytic remodeling [10, 39, 42]. Our data also align with prior observations in human beige adipocytes, where ISGylation of lactate dehydrogenase was linked to lower lactate production, recapitulated here in our system [42]. Moreover, the decreased ^13^C-labeling of pyruvate in our cells with active ISGylation mirrors the enhanced tracer incorporation in pyruvate seen in ISG15 knockout models [42]. Finally, our results agree with those of Bredow et al., who reported that ISGylation suppresses glycolysis in primary cardiomyocytes [10]. Their conclusions were based on functional assays measuring glycolytic rates and mitochondrial respiration, alongside in vitro enzyme assays indicating ISGylation inhibits key glycolytic regulators HK1 and PFK1. While our metabolomics data do not indicate modulation at these enzymes, given the accumulation of their intermediates, it does not preclude their inhibition, as such accumulation could also arise from a downstream bottleneck at the GAPDH step. Importantly, we did not detect ISG15 modification on HK1 or PFK1, which may reflect cell-type-specific differences in ISGylation targets or modification stoichiometry, and could also underlie this apparent discrepancy. Collectively, these observations highlight the role of cellular context in shaping the metabolic consequences of ISGylation and support a convergent model in which ISGylation limits glycolytic throughput.

Interestingly, while IFN treatment suppressed glycolysis, the effects were less pronounced compared to ISGylation alone. This aligns with studies reporting both negative and positive effects of type I interferons on glycolysis, suggesting that the outcome is context-dependent [6, 10, 39, 40, 60, 61]. Despite these discrepancies, a consistent observation across studies is that IFN treatment induces a stronger glycolytic response in ISG15 knockout cells or those expressing a non-conjugatable form of ISG15 compared to wild-type cells [10, 39], which supports the idea that ISG15 acts as a molecular brake on IFN-induced metabolic alterations. The relatively modest impact of IFN in our experiments may reflect buffering by other interferon-stimulated genes or shifts in substrate availability that obscure the specific contribution of ISGylation. Conceptually, modulating ISG15 levels or conjugation activity in the presence of IFN, or examining the temporal dynamics of glycolytic responses, could help clarify the extent to which ISGylation contributes to IFN-driven metabolic remodeling.

From an infectious disease perspective, ISGylation may help limit pathogen proliferation by restricting access to metabolic intermediates and energy, given that many pathogens rely on host metabolism for replication [62–67]. Supporting this notion, ISGylation has been shown to counteract coxsackievirus-induced upregulation of glycolysis, and modulate host metabolic responses to vaccinia virus and *Francisella novicida* infections [10, 40, 68]. Rather than directly targeting pathogen needs, ISGylation of glycolytic enzymes may also modulate the immune response to infections. For example, reduced lactate levels, through inhibition of LDHA activity, enhances type I IFN production and viral clearance in vivo [69], an interesting parallel given that ISGylation has previously been shown to reduce LDHA activity and lactate levels [42], further supported here. Together, these findings provide new insights into the metabolic role of ISGylation in antimicrobial defense, though further research in infection models will be needed to fully understand its broader relevance.

Lastly, our metabolomics analysis revealed that GAPDH represents a major site of glycolytic inhibition by ISGylation. Disruption at this node was marked by the accumulation of upstream substrates and depletion of downstream intermediates, consistent with a functional block in pathway progression. To establish whether this effect is directly mediated by ISGylation, we confirmed that GAPDH is modified by ISG15. Furthermore, in vitro enzyme activity assays demonstrated that ISGylation significantly impairs GAPDH catalytic activity, supporting the metabolic shift observed in our profiling. These findings position GAPDH as a primary regulatory target of ISGylation within the glycolytic pathway. While other enzymes such as HK1, PFK1, and LDHA have also been reported as ISGylation targets and may contribute to metabolic modulation, their influence appeared less pronounced in our experimental system [10, 42]. Moreover, the absence of comprehensive metabolomic profiling in earlier studies further complicates direct comparison of the relative contributions of these enzymes.

Nevertheless, ISG15 modification sites have been identified on nearly all glycolytic enzymes. Although the functional relevance of many of these modifications remains unclear, the breadth of targeting suggests a more coordinated regulatory mechanism. One explanation may lie in the co-translational model of ISGylation, which posits that proteins with high expression and translation efficiency, such as glycolytic enzymes, are more likely to be modified simply due to their abundance during synthesis [27, 36]. Yet, within this broadly targeted set, GAPDH stands out as particularly susceptible to ISGylation. Despite its efficient translation, GAPDH is among the slower-translated glycolytic enzymes and exhibits remarkably low protein turnover, based on publicly available ribosome profiling and proteome turnover data in HeLa cells [70, 71] (Additional file 1: Fig. S10). This suggests that once ISGylated, its modified pool persists for extended periods. Given the highly stable nature of ISGylation [72], this characteristic could result in long-term functional consequences, as newly synthesized unmodified GAPDH is replenished more slowly.

This raises the question of how persistent ISGylation impacts GAPDH activity. Our mutational analysis indicates that inhibition arises not from a single dominant site but from collective modification across multiple lysines, including residues close to the active site. Preventing ISGylation at only a few sites was insufficient to recover activity, whereas broader mutagenesis substantially restored function, demonstrating that multi-site modification underlies enzymatic inhibition. Importantly, this effect is not mediated by disruption of tetramer assembly as GAPDH still formed dimers and tetramers in cells with active ISGylation, including tetramers bearing ISG15. Rather, modification of interface-proximal and active-site–adjacent residues likely perturbs cooperative NAD⁺ binding and the conformational dynamics critical for catalytic efficiency in the GAPDH tetramer [57, 73]. In such a cooperative system, even partial modification, affecting only a subset of subunits, may be sufficient to dampen overall activity through dominant-negative effects, which provides a mechanistic basis for why GAPDH emerges as a prominent metabolic bottleneck in our dataset.

Finally, ISG15 modification may also indirectly interfere with GAPDH function by altering the intracellular distribution or compartmentalization of catalytically active GAPDH. In addition to its role in glycolysis, GAPDH has numerous moonlighting functions that are tightly regulated by post-translational modifications (PTMs), including phosphorylation, lysine acetylation, succination, arginine methylation, malonylation, palmitoylation, and redox-sensitive S-nitrosylation [52, 74]. Notably, several lysine residues known to regulate these functions overlap with ISG15 conjugation sites identified in our dataset and in other studies [31]. For instance, acetylation of K117, K227, and K251 promotes nuclear translocation of GAPDH during apoptotic stress [75]. ISGylation could either mimic or prevent such acetylation events, thereby disrupting the normal spatial distribution of GAPDH. Nuclear translocation of GAPDH is also regulated by a nuclear export signal (NES) located within its catalytic domain, which contains several lysine residues (KKVVKQASEGPLK), and mediates export via exportin-1 (CRM1) binding [76]. We and others found ISG15 conjugation sites within this region, raising the possibility that ISGylation may hinder CRM1-dependent export and lead to nuclear retention of GAPDH, especially considering that ISGylated proteins can accumulate in the nucleus [48, 77]. In addition to effects on localization, ISGylation may also directly impact GAPDH enzymatic function by targeting residues critical for catalytic regulation. For example, acetylation of K254 enhances GAPDH enzymatic activity, suggesting that ISGylation at this residue could attenuate its catalytic function [78]. Likewise, K219, a site involved in catalytic regulation and known to be deacetylated by rotavirus to enhance glycolysis, was also found to be ISGylated [79]. This indicates that ISGylation may compete with or shield against other regulatory PTMs, reducing GAPDH activity and potentially serving as a host defense mechanism against viral manipulation. Together, these findings suggest that ISGylation may also indirectly affect GAPDH function by influencing other PTMs that regulate its activity, further highlighting the complexity of GAPDH regulation beyond its catalytic role. Whether these effects extend to GAPDH’s broader cellular functions remains an important avenue for future investigation.

## Conclusions

This study provides new insight into how ISG15 shapes cellular metabolism through post-translational regulation. Using an integrated proteomics and metabolomics approach, we show that ISGylation modifies multiple glycolytic enzymes and imposes a metabolic checkpoint at GAPDH, where ISGylation decreases enzymatic activity. Mechanistic analyses indicate that this inhibition results from collective modification near GAPDH’s catalytic core, rather than disruption of its tetrameric assembly. Together, these findings establish GAPDH as a central metabolic effector of ISG15 and provide the first systems-level perspective on how ISGylation modulates central carbon metabolism.

## Methods

### Cell culture

HeLa cells (ATCC®-CCL-2) were purchased from ATCC (Rockville, MD, USA). HEK293T cells were described previously [28]. HeLa *ISG15*^−/−^ and parental wild-type cells were a kind gift of Prof. Dr. Antje Beling [38]. All cell lines were tested for mycoplasma infection prior to expansion in the laboratory. Material for cell culture, cell culture plastics and culture media were bought from ThermoFisher Scientific (Carlsbad, CA, USA). Cells were grown in Dulbecco’s Modified Eagle’s Medium (DMEM, 31966047, ThermoFisher Scientific) supplemented with 10% fetal bovine serum (FBS, #10270106, ThermoFisher Scientific). All cell cultures were maintained in a humidified incubator at 37 °C and 5% CO_2_.

### Plasmids and transfection

Cells were seeded and checked for ~ 80% confluency on the next day. Prior to transfection, medium was replaced with serum-free DMEM. Polyethylenimine (PEI, 23966–1, Polysciences) at a ratio of PEI/DNA 2.6:1 (w/w) was used as a transfection reagent to transfect the following plasmids: pSVsport-Mbu-v-17-mock, pMET7-Flag-eGFP, pcDNA3.1-HA-ISG15-LRGG (HA-ISG15), pcDNA3.1-HA-ISG15-LRAA (HA-ISG15-AA), pcDNA3.1-UBA7 (E1), pcDNA3.1-UBE2L6 (E2), pTriEx2-HERC5 (E3), pcDNA3.1-C-FLAG-GAPDH, pcDNA3.1-C-FLAG-GAPDH-K219R-K227R, pcDNA3.1-C-FLAG-GAPDH-13KR and pcDNA3.1-N-FLAG-PGK1. All aforementioned plasmids are listed in Table S6 (Additional file 2). Six hours post-transfection, the medium was replaced with DMEM supplemented with 10% fetal bovine serum. 

### Antibodies

The following primary antibodies, diluted at a final concentration of 1:1,000 in blocking buffer (LI-COR), were used for Western blot analysis: mouse anti-ISG15 antibody (F-9, Santa Cruz Biotechnology), rabbit anti-HA antibody (H6908, Sigma), mouse anti-FLAG antibody (F3165, Sigma-Aldrich), rabbit anti-GAPDH antibody (PA1-987, ThermoFisher Scientific), mouse anti-tubulin-α antibody (sc-5286, Santa Cruz Biotechnology) and rabbit anti-tubulin-α antibody (ab18251, Abcam). Secondary antibodies for Western blot analysis, diluted at a final concentration of 1:10,000 in blocking buffer (Li-COR), included: goat anti-mouse-IgG (IRDye® 800CW, LI-COR), goat anti-rabbit-IgG (IRDye® 800CW, LI-COR), goat anti-mouse-IgG (IRDye® 680RD, LI-COR) and goat anti-rabbit-IgG (IRDye® 680RD, LI-COR).

### SDS-PAGE and Western blot

Cleared cell lysates were prepared for SDS-PAGE in 4 × Laemmli Sample Buffer (Bio-Rad) supplemented with 100 mM dithiothreitol (DTT). Samples were sonicated by three pulses of 5 s at 40 V and heated at 95 °C for 10 min before loading onto 4–20% ExpressPlus™ PAGE Gels (M42015, GenScript). Approximately 20 µg of protein sample was loaded of each sample. In one lane, Precision Plus Protein™ Dual Color Standard (Bio-Rad) was loaded as a molecular weight standard. The gel was run for 68 min at 140 V in 1 × Tris-MOPS-SDS Running Buffer (M00138, GenScript) using the mini-PROTEAN Tetra Cell and PowerPac Basic Power Supply (#1658025, Bio-Rad). Proteins separated by SDS-PAGE were transferred to an Immobilon-P PVDF membrane (#IPFL00010, Merck) using the Criterion Blotter (#1656024, Bio-Rad) according to the manufacturer’s instructions. Following transfer at 100 V for 30 min in Tris/Boric buffer (50 mM Tris, 50 mM Boric acid), the membrane was blocked in blocking buffer (LI-COR) for 1 h at room temperature (RT). After blocking, the membrane was rinsed 3 times with demineralized water (ddH2O) followed by 1 wash of 5 min in TBS with 0.05% Tween (TBS-T) while shaking. Next, the membrane was incubated with primary antibody overnight at 4 °C. The following day, the membrane was rinsed three times with ddH2O followed by three washes of 5 min with TBS-T. After washing, the membrane was incubated with secondary antibody for 1 h at RT and washed two times in TBS-T and one time in TBS for 5 min each. Blots were imaged using an Odyssey Infrared Imaging System (LI-COR).

### ISGylomics sample preparation, LC–MS/MS and data analysis

5 million cells of each genotype (HeLa WT and *ISG15*^−/−^) were seeded in 150 mm plates (#639160, Greiner Bio-One) in triplicate for each condition (mock, IFN and ISG15). WT cells were either mock transfected or transfected with the ISGylation machinery (plasmids for E1, E2, E3, and HA-ISG15). *ISG15*^*−*/−^ cells were transfected in the same way, but excluding HA-ISG15 from the ISGylation machinery. For IFN conditions, mock-transfected cells were treated with 500 U/mL IFN-α2a (#11343504, ImmunoTools). After 48 h, cells were washed two times in PBS (#14040133, ThermoFisher Scientific), lysed in 5% SDS (50 mM TEAB pH 8.5) and boiled for 10 min at 95 degrees. Crude lysates were sonicated by three bursts of 5 s at 40 W followed by centrifugation at 10,000 g for 15 min at RT. The protein content of the cleared lysates was measured by BCA (#23225, Pierce) and 40 µg was set aside for Western blot analysis while 2 mg of total protein was prepared for MS analysis using the PTMScan HS Ubiquitin/SUMO Remnant Motif (K-ε-GG) Kit (#59322, Cell Signaling Technology). Briefly, proteins were reduced with DTT (4.5 mM) for 30 min at 55 °C prior to alkylation with chloroacetamide (10 mM) for 15 min at RT in the dark. Samples were subsequently acidified by adding 12% phosphoric acid and mixed with S-Trap Bind/Wash buffer (90% methanol, 100 mM TEAB pH 8.5) followed by immobilization on S-Trap midi columns (C02-mini-40, ProtiFi). Afterwards, proteins were digested on-column with 1:100 (w/w) trypsin (V5111, Promega) at 37 °C overnight. The next day, the resulting peptides were eluted from the column by adding S-Trap Elution Buffer A (50 mM TEAB pH 8.5), S-Trap Elution Buffer B (0.5% trifluoroacetic acid (TFA)) and S-Trap Elution Buffer C (50% ACN, 0.5% TFA), respectively, and centrifuging the column at 4,000 g for 1 min at RT in-between each step. Afterwards, the pH of the eluate was tested and adjusted if needed to pH < 3. At this point, 1% of the eluted peptides was taken for shotgun MS analysis. The remainder of the peptides was dried in a vacuum concentrator and re-dissolved in 1 × immunoprecipitation buffer before immunocapture of GG-modified peptides. The peptide solution was incubated with anti-K-ε-GG antibody bead slurry for 2 h at 4 °C on a rotator. Afterwards, beads were washed and GG-modified peptides were eluted by adding 0.15% TFA. Captured peptides were desalted on reversed-phase C18 OMIX tips (VARIA57003100K, Agilent Technologies), dried under vacuum in high-performance LC inserts and stored at −20 °C until further use.

Dried GG-modified peptides were redissolved in 33 µL of loading solvent A (0.1% TFA in water/ACN (98:2, v/v)) and 15 µL was injected for LC–MS/MS analysis on an Ultimate 3000 RSLCnano system connected in line to a Q Exactive HF mass spectrometer (ThermoFisher Scientific). Trapping was performed at 20 μL min^−1^ for 2 min in loading solvent A on a 5-mm trapping column (ThermoFisher scientific, 300 μm internal diameter (I.D.), 5 μm beads) and the sample was loaded on a 250-mm Aurora Series Emitter Column (IonOpticks, 1.6 µm C18 beads, 75 µm I.D.). Peptides were eluted by a nonlinear increase from 0 to 56% MS solvent B (0.1% formic acid (FA) in water/ACN (2:8, v/v)) over 135 min at a constant flow rate of 300 nL min^−1^, followed by a 15 min wash reaching 97% MS solvent B and re-equilibration with MS solvent A (0.1% FA in water/ACN (98:2, v/v)). The column temperature was kept constant at 50 °C in a column oven (CoControl 3.3.05, Sonation). The mass spectrometer was operated in data-dependent mode, automatically switching between MS and MS/MS acquisition for the eight most abundant ion peaks per MS spectrum. Full-scan MS spectra (375–1,500 m/z) were acquired at a resolution of 120,000 in the Orbitrap analyser after accumulation to a target value of 3,000,000. The 8 most intense ions above a threshold value of 8,300 were isolated for fragmentation at a normalized collision energy of 28% after filling the trap at a target value of 100,000 for a maximum of 120 ms. MS/MS spectra (200–2,000 m/z) were acquired at a resolution of 15,000 in the Orbitrap analyser. From the aliquots for shotgun proteomics analysis, approximately 1 µg of peptides were injected on the same LC–MS/MS system using similar settings as described above. The 16 most intense ions above a threshold value of 13,000 were isolated for fragmentation after filling the trap at a target value of 100,000 for a maximum of 80 ms.

All data were analyzed in FragPipe v21.1 using the LFQ-ubiquitin and LFQ-MBR workflow for the GG-peptidomics and shotgun analysis, respectively, with default settings including 1% FDR [80–84]. The spectra were searched against human protein sequences in the SwissProt database (database release version from January 2024 containing 20,644 human sequences). For GG-peptidomics searches, mass tolerance was set at a precursor mass tolerance of ± 10 ppm and fragment mass tolerance of 20 ppm. Digestion mode was set to ‘stricttrypsin’ enzyme specificity with up to three missed cleavages. Carbamidomethylation of cysteine residues was included as a fixed modification and oxidation of methionine, acetylation of protein N termini and GG-modification of lysine residues as variable modifications. The analysis led to the discovery of 15,619 GG-modified sites (listed in the combined_site_K_114.0429 table from FragPipe). For shotgun searches, mass tolerance was set at a precursor mass tolerance of ± 20 ppm and fragment mass tolerance of 20 ppm. Digestion mode was set to ‘stricttrypsin’ enzyme specificity with up to two missed cleavages. Carbamidomethylation of cysteine residues was included as a fixed modification and oxidation of methionine and acetylation of protein N as variable modifications. The shotgun analysis led to the discovery of 6,567 human proteins (listed in the combined_protein table from FragPipe).

GG-peptidomics data analysis was continued in Perseus v.1.6.2.1 after uploading the combined_site_K_114.0429 table from FragPipe. MaxLFQ intensities were log2-transformed and data was normalized through subtraction of the median intensity in each sample. Next, replicates were grouped and sites with less than three valid values in at least one replicate group were discarded. To allow statistical testing, missing data values were imputed from a normal distribution around the detection limit. The experimental design was uploaded into the site table by defining groups based on treatment (Mock, IFN, ISG15) and genotype (HeLa WT or *ISG15*^−/−^). A two-way analysis of variance (ANOVA) was done to compare the site intensities in the treatment group with the genotype group. Three p-values were calculated for each site, including a p-value for the effect of treatment and genotype and an interaction p-value. Sites with *p* < 0.01 for at least 1 of the 3 parameters were retained and used for unsupervised hierarchical clustering after z-score normalization. Significantly regulated sites were grouped into clusters and visualized in a heatmap in Fig. 1 with their intensity per replicate across the different experimental groups. The significantly regulated modification sites are listed in Table S1 (Additional file 2). In similar fashion, the shotgun proteomics data were further analyzed in Perseus after uploading the combined_protein table from FragPipe. MaxLFQ intensities were log2-transformed and the data were normalized by subtracting the median intensity in each sample. Replicates were grouped and proteins with fewer than three valid values in at least one replicate group were discarded. Missing data values were imputed from a normal distribution around the detection limit. To reveal proteins that were significantly regulated, samples were grouped in the same way as described above and a two-way ANOVA was performed to compare the maxLFQ intensities of the proteins in the treatment group with the genotype group. Proteins with p < 0.01 for at least 1 of the 3 parameters were considered to be significantly regulated. The maxLFQ intensities of these proteins after nonsupervised hierarchical clustering are shown in a heatmap in Fig. S4 (Additional file 1). The significantly regulated proteins are shown in Table S2 (Additional file 2). As only 2 major clusters were observed in the heatmap, a t-test was performed (FDR = 0.05 and S0 = 1) to compare protein intensities between all WT and *ISG15*^−/−^ samples. Quantified proteins (*n* = 4,873) and the results of the t-test are listed in Table S3 (Additional file 2) and shown in the volcano plot in Fig. S4 (Additional file 1).

### Tracer metabolomics sample preparation

0.5 million HeLa WT cells were seeded in quadruplicate in 6-well plates (#657160, Greiner Bio-One) for each condition (mock, IFN, and ISG15). Each condition included three biological replicates in C13 medium and one biological replicate in C12 control medium. Cells were either mock transfected or transfected with the ISG15 conjugation machinery (plasmids for E1, E2, E3, and HA-ISG15). For IFN conditions, mock-transfected cells were also treated with 500 U/mL IFN-α2a (#11343504, ImmunoTools). After 24 h, the medium was replaced with C13 labeled medium (DMEM (no glucose, no glutamine, no phenol red, A1443001, ThermoFisher Scientific) with 25 mM ^13^C_6_-glucose (VIB Metabolomics Core), 3.9723501 mM Glutamax (#35050061, ThermoFisher Scientific), 1 mM pyruvate (#11360070, ThermoFisher Scientific) and 10% FBS) or C12 unlabeled medium (DMEM (no glucose, no glutamine, no phenol red, A1443001, ThermoFisher Scientific) with 25 mM 12C-glucose (VIB Metabolomics Core), 3.9723501 mM Glutamax, 1 mM pyruvate and 10% FBS). For the IFN conditions, additional IFN-α2a (500 U/mL) was added to the medium to keep the IFN stimulation consistent. Twenty-four hours later, cells were washed once with ice cold (4 °C) 0.9% NaCl solution and lysed in methanol extraction buffer (80% methanol and 2 µM d27 myristic acid) precooled at −80 °C. After centrifugation at 20,000 g for 15 min at 4 °C, supernatants containing the metabolites were stored at −80 °C until further use, while the protein pellet was dissolved in 5% SDS and set aside for Western blot analysis.

### LC–MS and metabolomics data analysis

Metabolite samples were analyzed by the VIB Metabolomics Core according to in-house protocols. Briefly, 10 µL of each sample was loaded onto a Dionex UltiMate 3000 LC System (ThermoFisher Scientific) equipped with a C18 column (Acquity UPLC-HSS T3 1.8 µm, 2.1 × 150 mm, Waters) coupled to a Q Exactive Orbitrap mass spectrometer (ThermoFisher Scientific) operating in negative ion mode. A step gradient was carried out using solvent A (10 mM tributylamine (TBA) and 15 mM acetic acid) and solvent B (100% methanol). The gradient started with 5% of solvent B and 95% solvent A and remained at 5% B until 2 min post injection. A linear gradient to 37% B was carried out until 7 min and increased to 41% until 14 min. Between 14 and 26 min the gradient increased to 95% of B and remained at 95% B for 4 min. At 30 min the gradient returned to 5% B. The chromatography was stopped at 40 min. The flow was kept constant at 0.25 mL/min and the column was placed at 40 °C throughout the analysis. The mass spectrometer was operated in full scan mode (m/z range: [70.0000–1050.0000]) using a spray voltage of 4.80 kV, capillary temperature of 300 °C, sheath gas at 40.0 and auxiliary gas at 10.0. The AGC target was set at 3,000,000 using a resolution of 140,000, with a maximum IT fill time of 512 ms. Data collection was performed using the Xcalibur software (ThermoFisher Scientific). The data analyses were performed by integrating the peak areas (El-Maven – Polly—Elucidata) [85]. Raw abundances and fractional contributions, detailed in Table S4 (Additional file 2), were analyzed and visualized in TraVisPies as described previously [49].

### Immunoprecipitation (IP) and ISG15 target validation

HEK293T cells were grown at ~ 80% confluence in 150 mm plates (#639160, Greiner Bio-One). Cells were transfected either with FLAG-GFP, FLAG-GAPDH or FLAG-PGK1 plasmids, plus, the ISG15 conjugation machinery (E1, E2, E3 and HA-ISG15). After 48 h, cells were washed three times in PBS and scraped in 1.5 mL IP lysis buffer (50 mM Tris–HCl pH 8.0, 150 mM NaCl, 1% Triton-X-100 (v/v), 1 mM PMSF (#10837091001, Roche) and 1 × protease inhibitor cocktail (#4693159001, Roche)). Samples were incubated for 20 min by end-over-end agitation at 4 °C. Lysates were cleared by centrifugation for 15 min at 16,000 g at 4 °C to remove insoluble components. Supernatants were incubated with anti-FLAG magnetic beads (#M8823, Merck) at 4 °C under agitation for 2 h. The beads were precipitated with a magnetic stand and subsequently washed five times in IP wash buffer (50 mM Tris–HCl pH 8.0, 150 mM NaCl and 1% Triton-X-100 (v/v)). Afterwards, beads were mixed with 60 µL 2 × Laemmli Sample Buffer and heated at 60 °C for 10 min. Beads were precipitated with a magnet and the supernatants were analyzed by Western blot.

### AP-MS sample preparation, LC–MS/MS and data analysis

HEK293T cells were grown at ~ 80% confluence in 100 mm plates (#664160, Greiner Bio-One). Cells were transfected with plasmids encoding the ISG15 conjugation enzymes (E1, E2 and E3), HA-ISG15 or HA-ISG15-AA, and FLAG-GFP, FLAG-GAPDH or FLAG-PGK1. After 48 h, cells were washed three times in PBS and lysed in 0.3 mL AP-MS lysis buffer (50 mM Tris–HCl pH 8.0, 150 mM NaCl, 0.5% NP-40 (v/v), 1 mM PMSF and 1 × protease inhibitor cocktail). Samples were incubated for 20 min by end-over-end agitation at 4 °C. Lysates were cleared by centrifugation for 15 min at 16,000 g at 4 °C to remove insoluble components. Supernatants were diluted 1:5 in AP-MS lysis buffer without NP-40 and incubated with anti-FLAG magnetic beads (#M8823, Merck) at 4 °C under agitation for 2 h. The beads were precipitated with a magnetic stand and subsequently washed three times in AP-MS wash buffer (50 mM Tris–HCl pH 8.0, 150 mM NaCl and 0.1% NP-40 (v/v)) and three times in trypsin digestion buffer (20 mM Tris–HCl pH 8.0, 2 mM CaCl_2_). Next, proteins were digested on-bead with 1 µg trypsin (V5111, Promega) at 37 °C for 4 h while shaking. After magnetic separation, the supernatant was collected and digested overnight at 37 °C with an additional 1 µg trypsin. The samples were acidified to 1% TFA and desalted on reversed-phase C18 OMIX tips (VARIA57003100K, Agilent Technologies), dried under vacuum in high-performance LC inserts and stored at − 20 °C until further use.

Peptides were redissolved in 20 µL of loading solvent A, and 2 µL was injected for LC–MS/MS analysis. The separation and detection were performed on an Ultimate 3000 RSLCnano system connected to a Q Exactive HF mass spectrometer. Trapping was performed at 10 μL/min for 4 min in loading solvent A on a 20 mm trapping column (made in-house, 100 μm internal diameter (I.D.), 5 μm beads, C18 Reprosil-HD, Dr. Maisch, Germany). The sample was loaded on an in-house produced 250 mm analytical column (75 µm I.D.), equipped with a laser pulled electrospray tip using a P-2000 Laser Based Micropipette Puller (Sutter Instruments), packed in-needle with ReproSil-Pur basic 1.9 µm silica particles (Dr. Maisch). Peptide elution was performed over 135 min as described above. The mass spectrometer was operated in data-independent mode, automatically switching between MS and MS/MS acquisition. Full-scan MS spectra ranging from 375–1500 m/z with a target value of 5,000,000, a maximum fill time of 50 ms and a resolution at of 60,000 were followed by 30 quadrupole isolations with a precursor isolation width of 10 m/z for HCD fragmentation at an NCE of 30% after filling the trap at a target value of 3,000,000 for maximum injection time of 45 ms. MS/MS spectra (400–900 m/z) were acquired at a resolution of 15,000 in the Orbitrap analyser.

Data were analyzed in DIA-NN version 1.9.0 with default search settings and a false discovery rate set at 1% on the precursor and protein level [86]. The spectra were searched against human protein sequences in the SwissProt database, including GFP (database release version from January 2024 containing 20,644 human sequences). Mass tolerance was set at a precursor mass tolerance of ± 10 ppm and fragment mass tolerance of 20 ppm. Digestion mode was set to ‘Trypsin/P’ enzyme specificity with up to two missed cleavages. Carbamidomethylation of cysteine residues was included as a fixed modification and oxidation of methionine, acetylation of protein N termini, and GG-modification of lysine residues as variable modifications. The analysis led to the discovery of 26 GG-modified sites (listed in the report.UniMod_121_sites_99 table from DIA-NN).

Data analysis was continued in Perseus v.1.6.2.1 after uploading the report.UniMod_121_sites_99 table. MaxLFQ intensities were log2-transformed and data was normalized through subtraction of the median intensity in each sample. Next, replicates were grouped and sites with less than three valid values in at least one replicate group were discarded. To allow statistical testing, missing data values were imputed from a normal distribution around the detection limit. Site intensities between each experimental group were compared using a one-way ANOVA. Sites with *p* < 0.01 were retained and used for unsupervised hierarchical clustering after z-score normalization. Significantly regulated sites were grouped into clusters and sites on GAPDH and PGK1 were visualized in a heatmap in Fig. S7 (Additional file 1) with their intensity per replicate across the different experimental groups.

### Enzyme activity assays

HEK293T cells were grown at ~ 80% confluence in 100 mm plates (#664160, Greiner Bio-One). Cells were transfected with plasmids encoding the ISG15 conjugation enzymes (E1, E2 and E3), HA-ISG15 or HA-ISG15-AA, wild-type or KR mutant FLAG-GAPDH, or FLAG-PGK1. After 48 h, cell lysates were prepared and subjected to immunoprecipitation as described above. GAPDH activity was evaluated using a commercial enzyme assay kit (ab204732, Abcam). Briefly, beads containing immunoprecipitated GAPDH were resuspended in 100 µL IP wash buffer and 1 µL of beads was added to a reaction mix containing GAPDH assay buffer, GAPDH developer and GAPDH substrate. The absorbance was measured at 450 nm using a plate reader (VarioSkan LUX, ThermoFisher Scientific) after 10 min incubation at 37 °C. Similarly, PGK1 activity was measured using a commercial enzyme assay kit following the manufacterer’s instructions (ab252890, Abcam). Recorded activities were normalized to the immunoprecipitated protein levels and expressed relative to baseline across three independent biological repeats. Data were analyzed in GraphPad Prism 10.3.1.

### DTSSP crosslinking

HeLa and HEK293T cells were grown at ~ 80% confluence in 100 mm plates. HeLa cells were transfected with mock plasmids or plasmids encoding the ISG15 conjugation enzymes (E1, E2, E3) and HA-ISG15 or HA-ISG15-AA. HEK293T cells were transfected with plasmids encoding the ISG15 conjugation enzymes (E1, E2 and E3), HA-ISG15 or HA-ISG15-AA, and FLAG-GAPDH. HeLa cells were lysed in crosslinking lysis buffer (PBS, pH 7.4, 1% Triton X-100 (v/v), 1 mM PMSF, and 1 × protease inhibitor cocktail). HEK293T lysates were additionally subjected to anti-FLAG IP, and bound proteins were eluted with 340 µM 3 × FLAG peptide (SB045, SB-PEPTIDE) for 30 min at 4 °C with shaking (1200 rpm). For crosslinking, samples were incubated with 2 mM 3,3′-dithiobis(sulfosuccinimidyl propionate) (DTSSP) for 30 min at 4 °C with shaking (700 rpm). Reactions were quenched by addition of 100 mM Tris (pH 8.0) for 10 min at 4 °C with shaking (700 rpm). Samples were mixed with 4 × Laemmli buffer and divided into reducing (100 mM DTT) and non-reducing aliquots before Western blot analysis.

## Supplementary Information

Additional file 1: Figs. S1-10. Fig. S1: Establishing a cellular ISGylation model. Fig. S2: Validation of ISGylation induction in proteomics samples. Fig. S3: ISG15 sites specifically upregulated upon ectopic expression of the ISGylation machinery. Fig. S4: Proteome analysis of wild-type and ISG15 KO HeLa cells. Fig. S5: PCA of glycolytic and TCA cycle metabolites. Fig. S6: Limited tracer incorporation and subtle metabolic changes in the TCA cycle of HeLa cells with active ISGylation. Fig. S7: AP-MS confirms ISG15 modification of GAPDH and PGK1. Fig. S8: ISGylation does not affect GAPDH oligomerization. Fig. S9: Structural mapping of ISG15 sites on PGK1. Fig. S10: Protein half-life and translation efficiency of glycolytic enzymes in HeLa cells.

Additional file 2: Tables S1-6. Table S1. List of GlyGly(K) sites derived from ISG15 (cluster 1) or ubiquitin (cluster 2-5) obtained after two-way ANOVA and hierarchical clustering. Table S2. List of proteins derived from the protein heatmap obtained after two-way ANOVA and hierarchical clustering. Table S3. List of quantified proteins after t-testing. Table S4. Supporting data for the metabolomics analyses presented in this study. Table S5. ISG15 sites on GAPDH and PGK1 identified across different studies. Table S6. Plasmid information.

Additional file 3. Uncropped scans of all Western blots presented in this study.

## Data Availability

The mass spectrometry proteomics data have been deposited to the ProteomeXchange Consortium via the PRIDE [87] partner repository with the dataset identifiers PXD065158 [88] and PXD071724 [89]. The metabolomics data have been deposited to the MetaboLights repository [90] under the study identifier MTBLS12619 [91]. Published sequencing data were obtained from the NCBI Gene Expression Omnibus under accession number GSE22004 [70, 92].
