# Supplementary material for: Proteomics and tracer metabolomics link GAPDH ISGylation to glycolytic control
Source: Genome Biol. 2026 Mar 11;27:135. doi: 10.1186/s13059-026-04034-w (PMC13093933; doi:10.1186/s13059-026-04034-w)
Supplement: Supplementary file 1 — Additional file 1: Figs. S1-10. Fig. S1: Establishing a cellular ISGylation model. Fig. S2: Validation of ISGylation induction in proteomics samples. Fig. S3: ISG15 sites specifically upregulated upon ectopic expression of the ISGylation machinery. Fig. S4: Proteome analysis of wild-type and ISG15 KO HeLa cells. Fig. S5: PCA of glycolytic and TCA cycle metabolites. Fig. S6: Limited tracer incorporation and subtle metabolic changes in the TCA cycle of HeLa cells with active ISGylation. Fig. S7: AP-MS confirms ISG15 modification of GAPDH and PGK1. Fig. S8: ISGylation does not affect GAPDH oligomerization. Fig. S9: Structural mapping of ISG15 sites on PGK1. Fig. S10: Protein half-life and translation efficiency of glycolytic enzymes in HeLa cells. [file 13059_2026_4034_MOESM1_ESM.pdf]

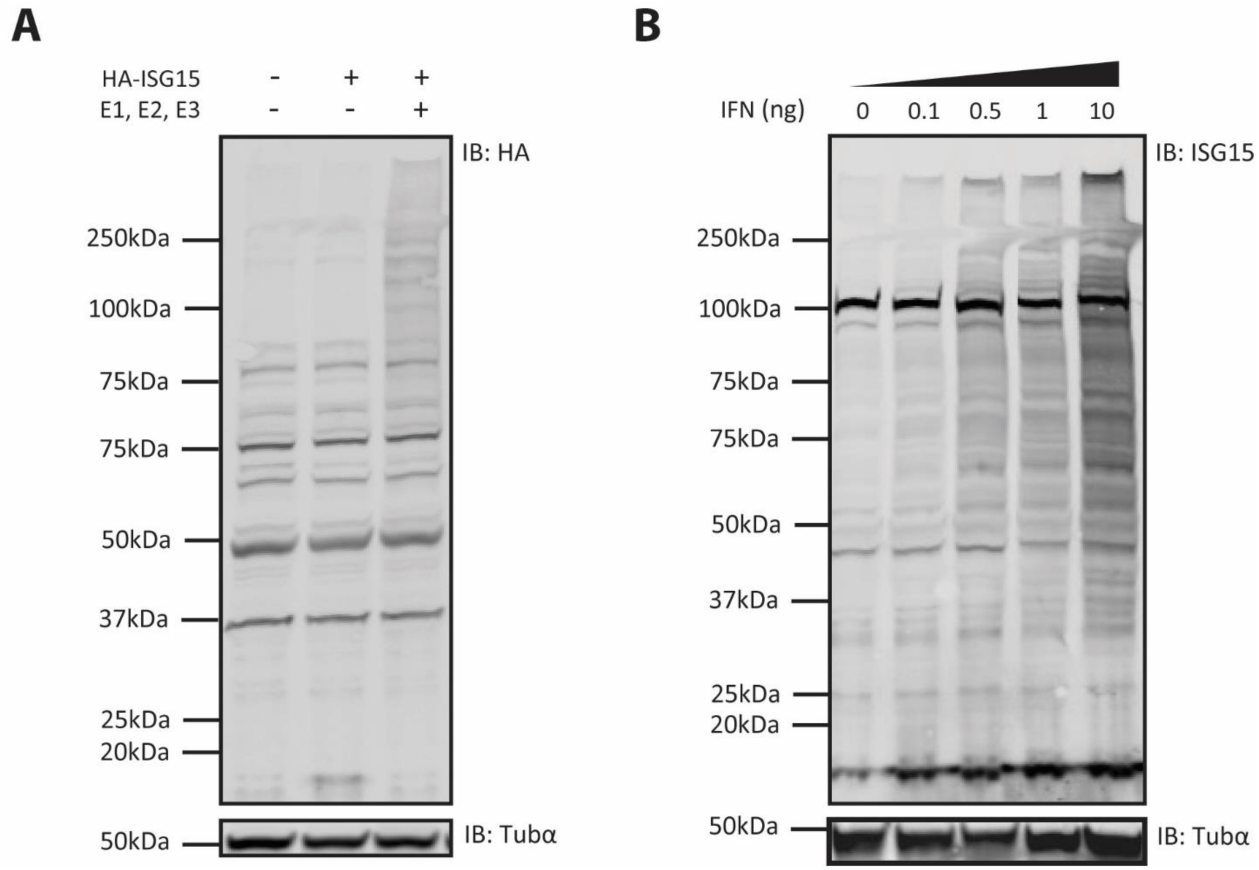

Fig. S1: Establishing a cellular ISGylation model. (A) HeLa cells were transfected with plasmids encoding the ISGylation machinery (E1, E2, E3 and HA-ISG15). After 48 h, cells were lysed and analyzed by immunoblotting using an anti-HA antibody (IB: HA). Expression of HA-ISG15 alone resulted in a single band corresponding to free ISG15 (~15 kDa), while co-expression with the full conjugation machinery produced a high-molecular-weight smear representing ISG15-conjugated proteins. Tubulin was used as loading control (IB: Tubα). **(B)** HeLa cells were treated with increasing concentrations of IFN (0, 0.1, 0.5, 1, or 10 ng/mL) for 48 hours to induce endogenous ISGylation. Immunoblotting with an anti-ISG15 antibody (IB: ISG15) showed a dose-dependent increase in both free and conjugated ISG15 species. α-Tubulin was used as loading control (IB: Tubα).

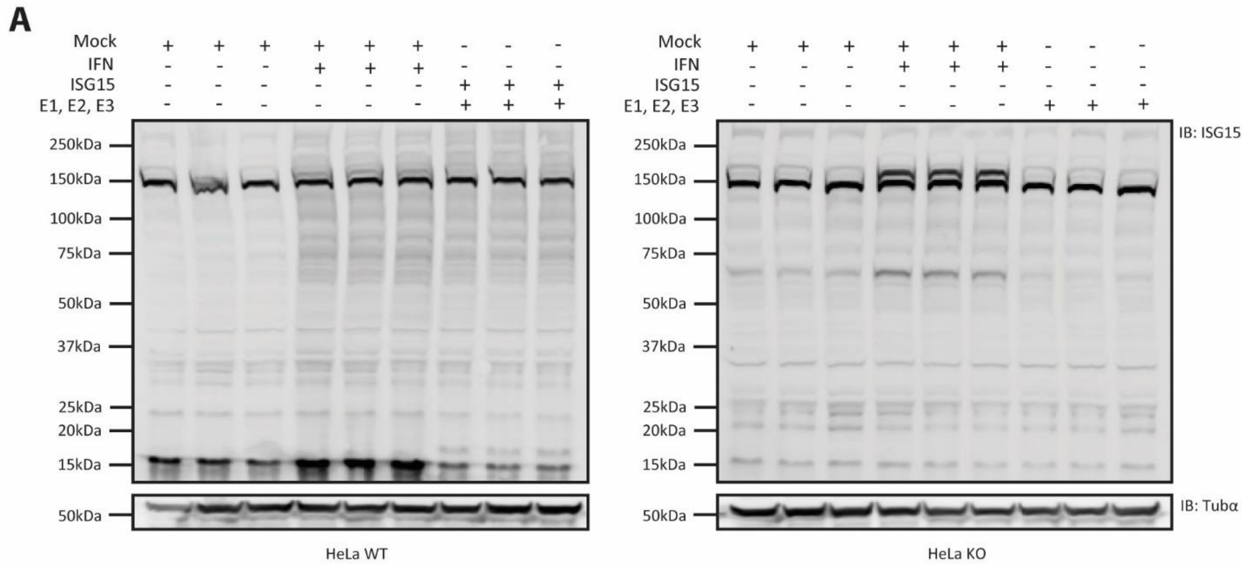

Fig. S2: Validation of ISGylation induction in proteomics samples. (Left) HeLa wild-type (WT) cells were transfected with either a mock plasmid, the ISGylation machinery (E1, E2, E3, and HA-ISG15), or treated with IFN in combination with mock transfection. (Right) HeLa *ISG15*$^{-/-}$ (KO) cells were transfected with either a mock plasmid, the ISGylation machinery excluding HA-ISG15 (E1, E2, E3), or treated with IFN and mock-transfected. ISG15 conjugates were detected by immunoblotting using an anti-ISG15 antibody (IB: ISG15). Free ISG15 appears as a single band (~15 kDa). α-Tubulin was included as a loading control (IB: Tubα).

A

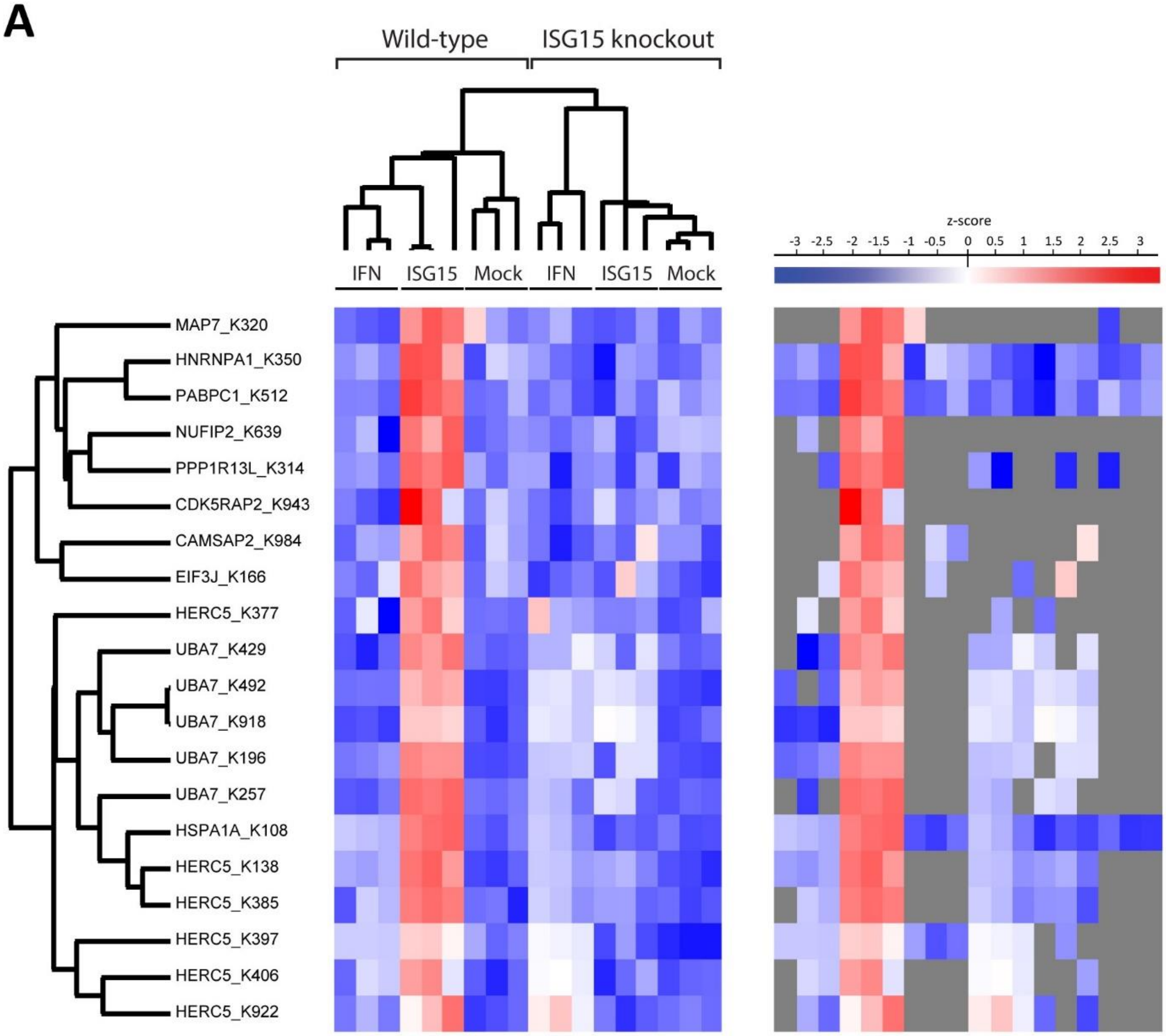

Fig. S3: ISG15 sites specifically upregulated upon ectopic expression of the ISGylation machinery. Heatmap detail of cluster 1a showing GlyGly(K) sites specifically upregulated upon ectopic expression of the ISGylation machinery. Shown is a magnified view of the corresponding region from the full heatmap displayed in Fig. 1B. Different genotypes (wild-type or *ISG15* KO) and treatments (IFN, ISG15, or Mock) are indicated. The colors represent upregulated (red), downregulated (blue) or missing (gray) sites.

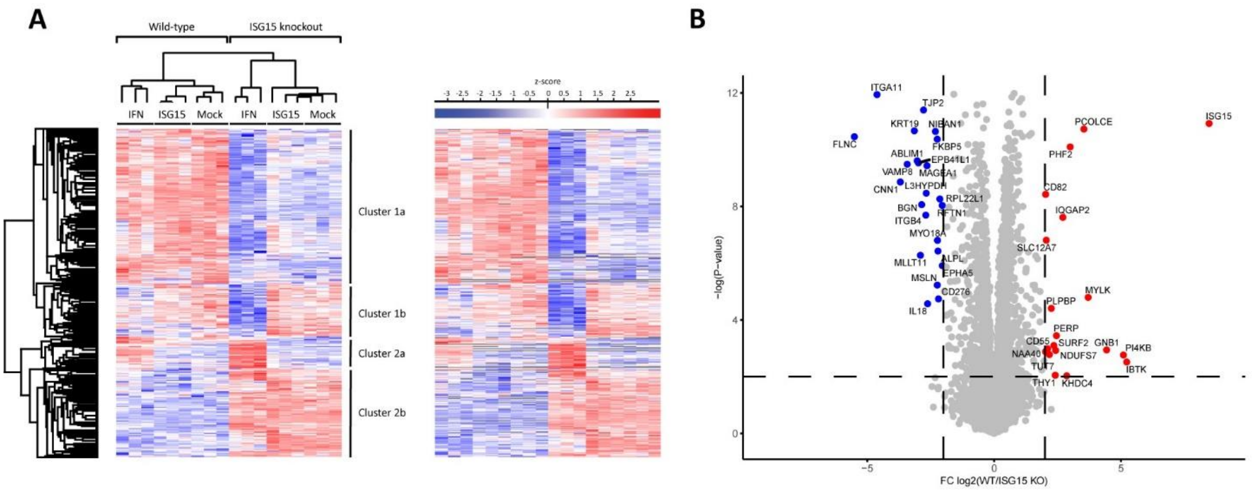

Fig. S4: Proteome analysis of wild-type and *ISG15* KO HeLa cells. (A) Heatmap showing significantly regulated proteins identified by shotgun proteomics following IFN stimulation or overexpression of the ISGylation machinery in wild-type (WT) and *ISG15* knockout (KO) HeLa cells. Unsupervised hierarchical clustering was performed across all samples and conditions: mock-treated, IFN-stimulated, or ISG15 machinery-overexpressing (ISG15). Protein expression changes are represented by color intensity (red: upregulated; blue: downregulated). The right panel displays the same heatmap with missing values shown in gray. Two major clusters can be observed corresponding to proteins that are up- or downregulated in WT versus *ISG15* KO cells, listed in Table S2 (Additional file 2). **(B)** To compare proteome-wide differences between genotypes, a t-test was performed (FDR = 0.05, S0 = 1) using all WT (n = 9) and *ISG15* KO (n = 9) samples. The volcano plot shows log2 fold changes (WT vs. KO) on the x-axis and statistical significance (–log P value) on the y-axis. A total of 120 proteins were significantly upregulated and 126 downregulated in WT cells compared to *ISG15* KO cells. Quantified proteins and statistical results are listed in Table S3 (Additional file 2).

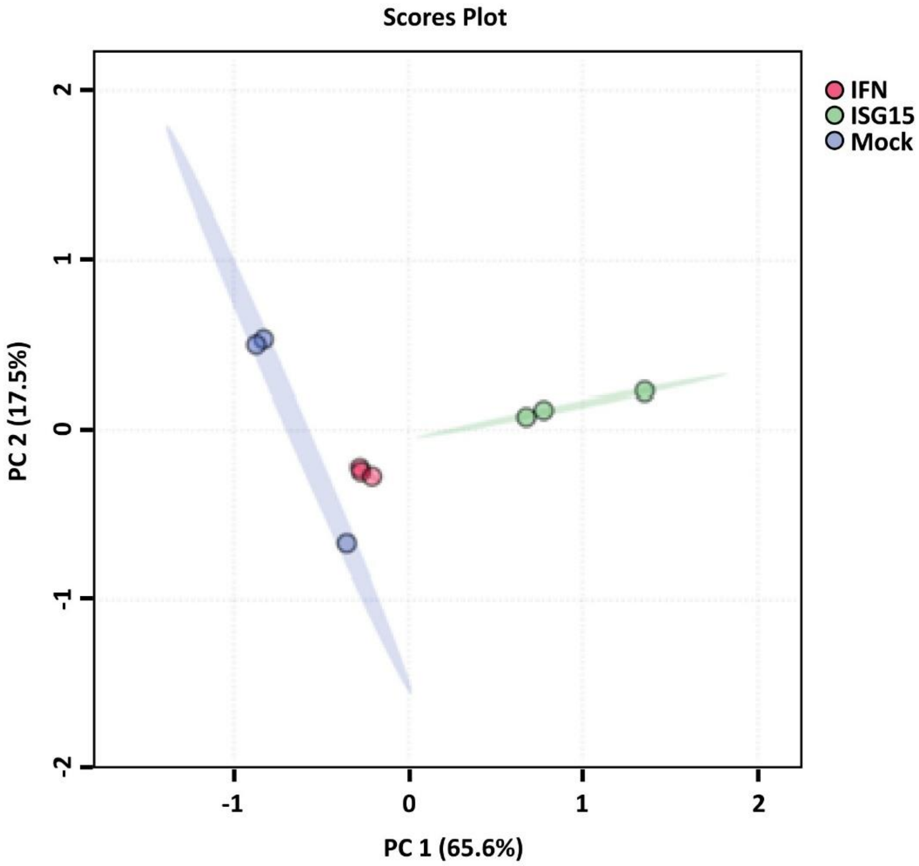

Fig. S5: PCA of glycolytic and TCA cycle metabolites. Principal component analysis (PCA) was performed on metabolomics data from HeLa cells treated under three conditions: mock-transfected (blue), IFN-treated (red), or transfected with the ISGylation machinery (E1, E2, E3, and ISG15; green). Each dot represents an individual sample, and ellipses indicate the 95% confidence intervals for each group. The plot was generated after uploading and processing the data on MetaboAnalyst 5.0 (Additional file 2: Table S4).

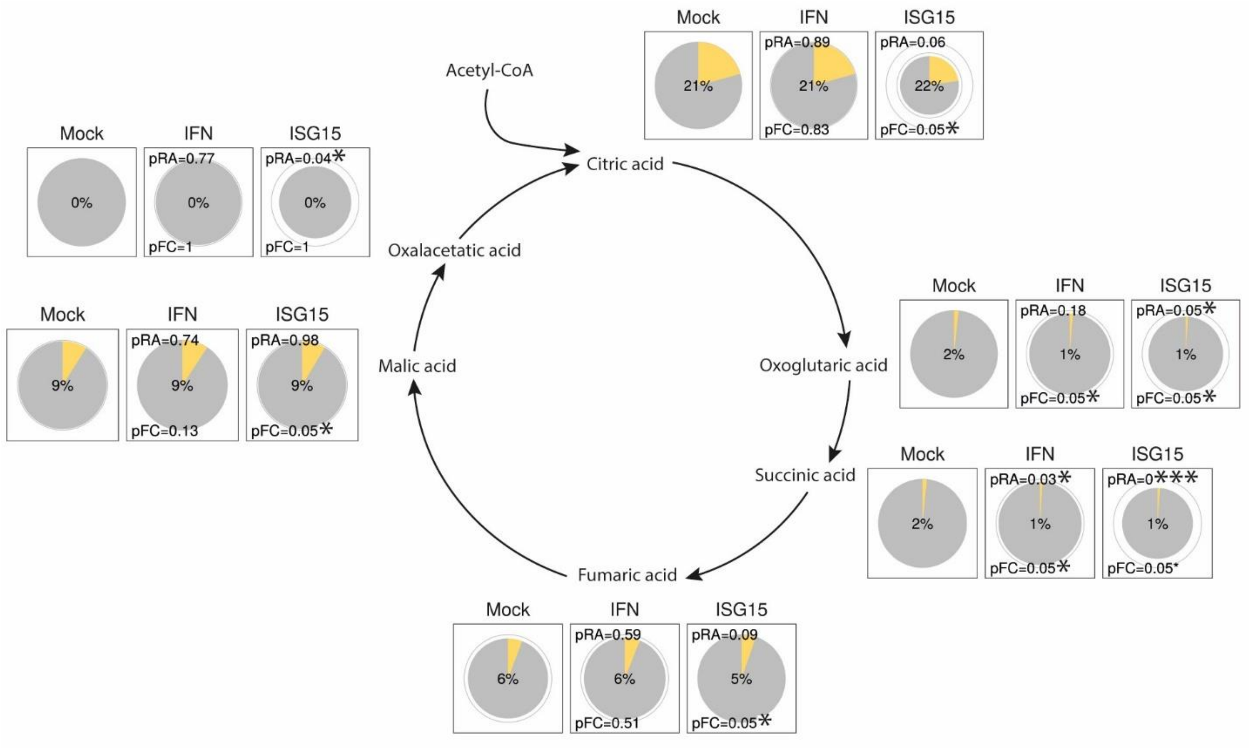

Fig. S6: Limited tracer incorporation and subtle metabolic changes in the TCA cycle of HeLa cells with active ISGylation. TraVis pie visualizations of the metabolomics data mapped onto the tricarboxylic acid (TCA) cycle, summarizing metabolite abundance and $^{13}C_6$-glucose-derived labeling across three conditions: mock-transfected controls (Mock), IFN-treated cells (IFN), and cells transfected with the ISGylation machinery (ISG15). Pie radii represent relative metabolite abundance, while yellow segments and central percentages indicate the fractional contribution from labeled glucose. The remaining segment (gray) represents the fractional contribution from unlabeled sources. Statistical significance for relative abundance (pRA) and fractional contribution (pFC) is shown relative to the mock condition. Comparisons were made using two-tailed Student's t-tests (n = 3 independent biological repeats). * $p<0.05$, ** $p<0.01$, *** $p<0.001$.

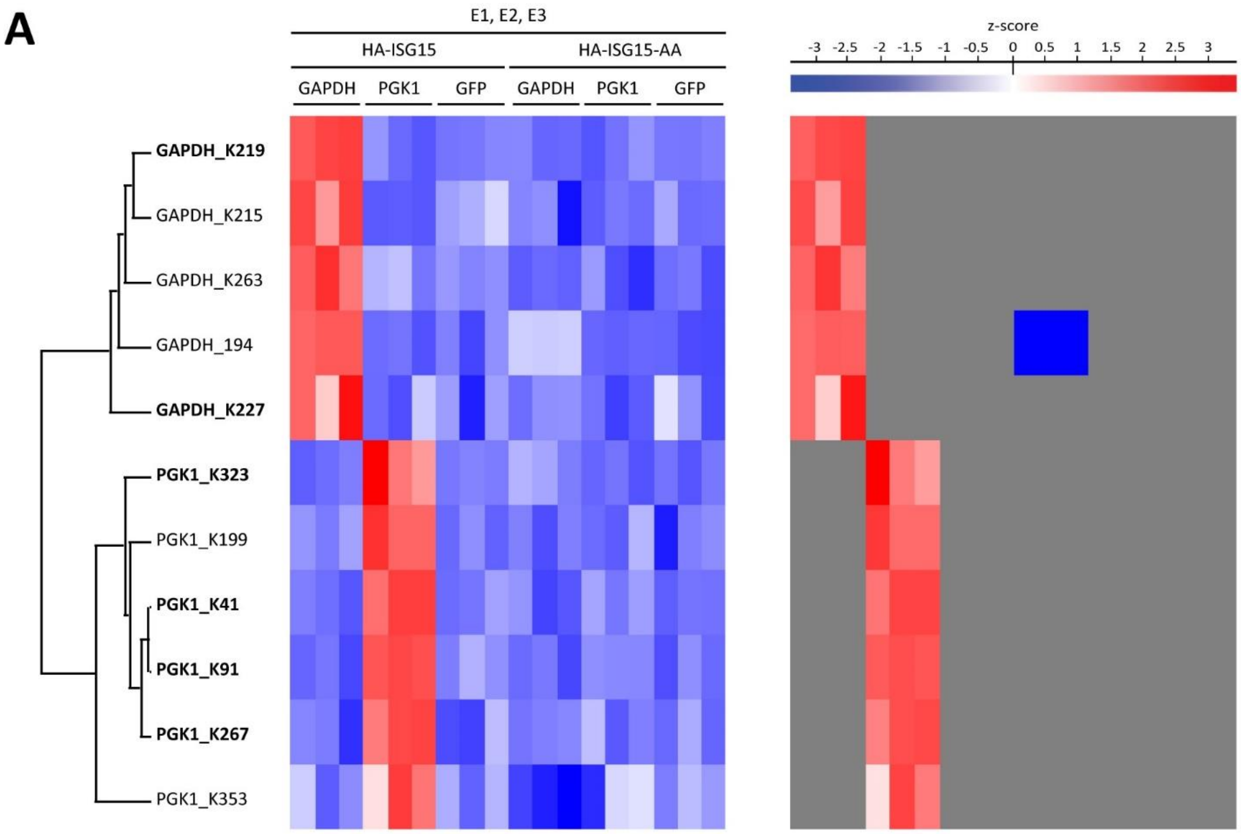

Fig. S7: AP-MS confirms ISG15 modification of GAPDH and PGK1. Left, heatmap of significantly regulated GlyGly(K) sites on GAPDH and PGK1 identified by AP-MS following immunoprecipitation of FLAG-GAPDH, FLAG-PGK1 or FLAG-GFP from HEK293T cells expressing the ISGylation machinery (E1, E2, E3) with either wild-type HA-ISG15 or the conjugation-defective mutant HA-ISG15-AA (n = 3 independent biological repeats). Sites are defined as ISG15-specific if significantly enriched in the presence of HA-ISG15 compared to HA-ISG15-AA. The colors represent upregulated (red) or downregulated (blue) sites. Right, the same heatmap is shown with the originally missing values depicted in gray. Sites indicated in bold were also detected in our ISGylomics dataset.

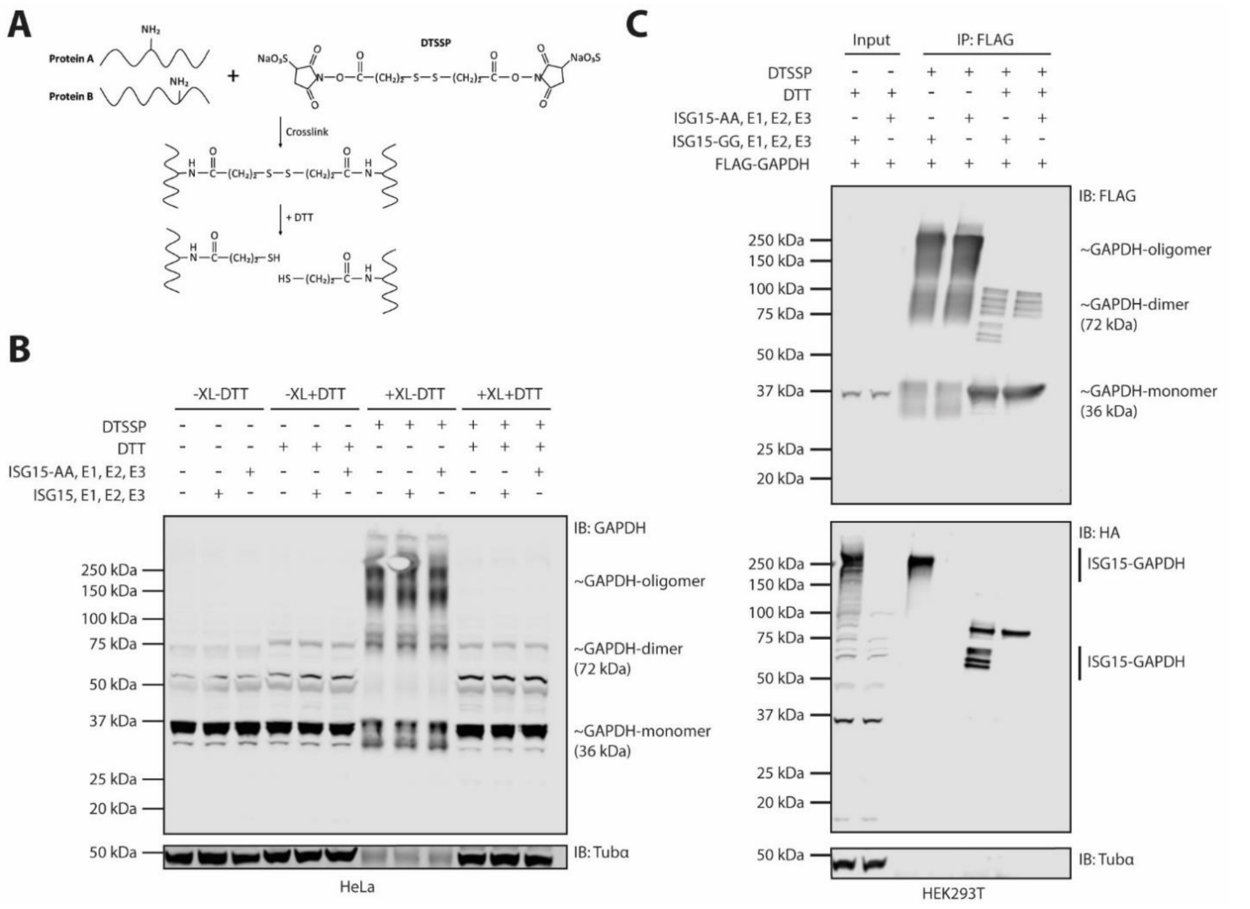

Fig. S8: ISGylation does not affect GAPDH oligomerization. (A) Schematic of the 3,3'-dithiobis(sulfosuccinimidyl propionate) (DTSSP) crosslinking reaction. DTSSP is a reduction-sensitive protein crosslinker that covalently links proteins via primary amines. The disulfide bridge within DTSSP allows crosslinks to be reversed by treatment with a reducing agent such as dithiothreitol (DTT). **(B)** HeLa cells were transfected with either mock plasmids or the ISG15 conjugation enzymes (E1, E2, E3) together with wild-type HA-ISG15 or the conjugation-defective HA-ISG15-AA mutant. Cells were lysed, and proteins in the lysate were subjected to DTSSP crosslinking or left untreated. Crosslinks were reversed in a subset of samples by DTT treatment, and all samples were analyzed by immunoblot. Membranes were probed with anti-GAPDH to detect oligomers (IB: GAPDH) and anti-α-tubulin as a loading control (IB: Tubα). GAPDH oligomers are indicated. **(C)** FLAG-tagged GAPDH was immunoprecipitated from HEK293T cells co-expressing HA-ISG15 or HA-ISG15-AA together with the ISG15 conjugation enzymes. Bound proteins were eluted with 3×FLAG peptide and crosslinked with DTSSP. Samples were split, treated

with or without DTT to reverse the crosslinks, and analyzed by immunoblot. Membranes were probed with anti-FLAG to detect GAPDH oligomers, anti-HA to confirm conjugation (IB: HA), and anti-α-tubulin as a loading control (IB: Tubα). GAPDH oligomers are indicated.

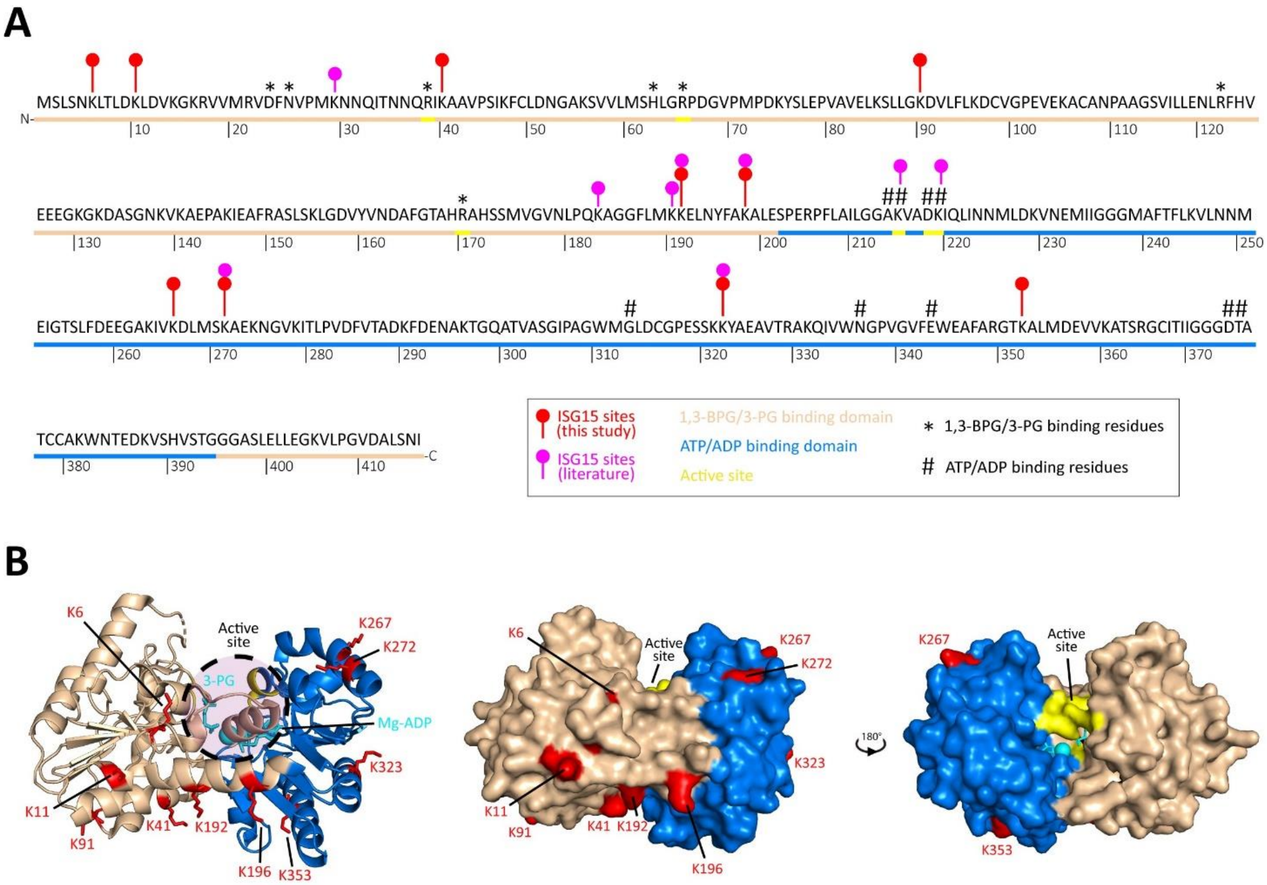

Fig. S9: Structural mapping of ISG15 sites on PGK1. (A) ISG15 modification sites identified in this study (red) and recurrently detected in other studies (magenta) are mapped onto the amino acid sequence of wild-type human PGK1 (Additional file 2: Table S5) [10, 31, 36, 38, 54]. The domain organization of PGK1 is shown, with the 1,3-biphosphoglycerate (BPG)/3-phosphoglycerate (PG)-binding domain (residues 1-202 and 396-417) colored beige, the ATP/ADP binding domain (residues 203-395) colored blue and the active site residues in yellow. Residues involved in 1,3-BPG/3-PG binding (*) and ATP/ADP binding (#) are annotated above the sequence [59]. **(B)** PGK1 is shown with the identified ISG15 modification sites highlighted in red (stick representation), displayed in both cartoon (left) and surface (right) views (PDB 2X13). The 1,3-BPG/3-PG-binding domain is colored beige, the ATP/ADP binding domain blue, and the active site in yellow. An Mg-ADP and 3-PG molecule bound to the subunit are shown in cyan (stick representation). Models were created using PyMOL Molecular Graphics System v3.1.3 (Schrödinger, LLC).

A

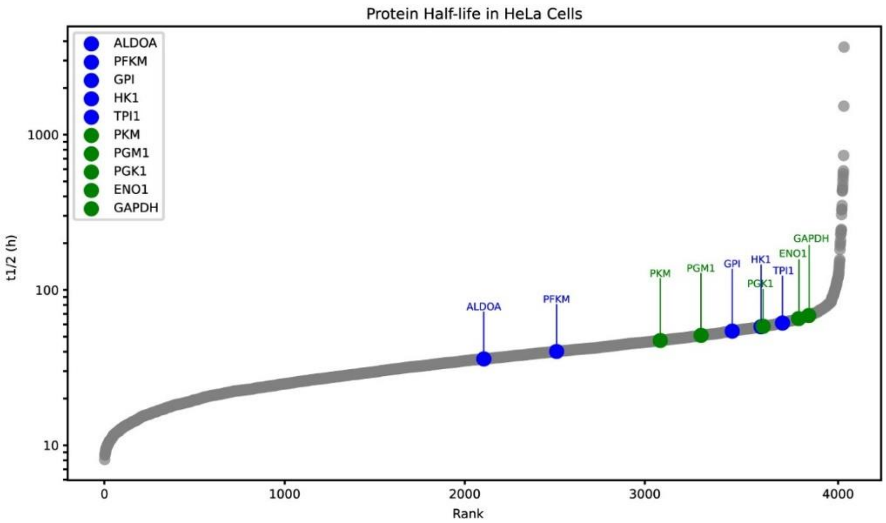

B

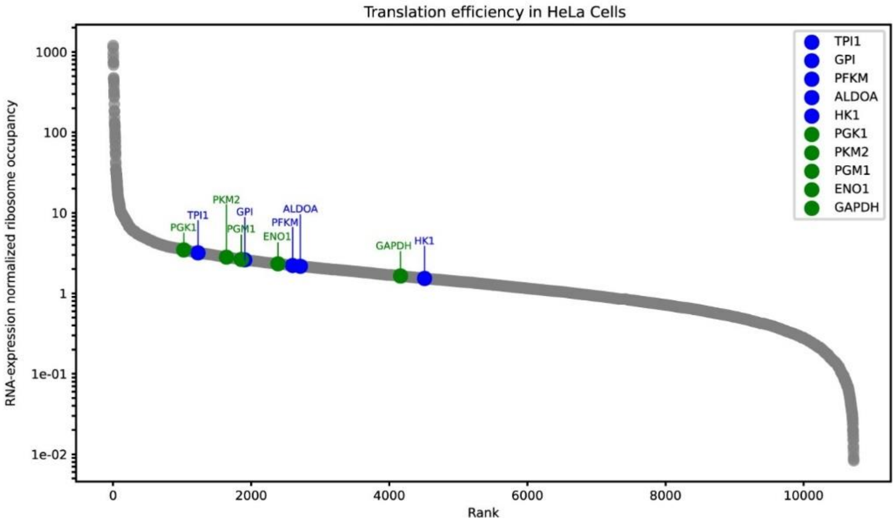

Fig. S10: Protein half-life and translation efficiency of glycolytic enzymes in HeLa cells. Processed data from previously published studies were used to obtain protein half-life **(A)** and translation efficiency **(B)** for HeLa cell proteins [70, 71]. In each case, proteins were ranked: shorter half-lives received higher ranks in (A), and higher translation efficiencies received higher ranks in (B). The distribution of glycolytic enzymes across ranks is shown for each dataset. Enzymes involved in the energy-investment phase of glycolysis are shown in blue, while those in the energy-payoff phase are shown in green.
