# Supplementary material for: Proteomics and tracer metabolomics link GAPDH ISGylation to glycolytic control
Source: Genome Biol. 2026 Mar 11;27:135. doi: 10.1186/s13059-026-04034-w (PMC13093933; doi:10.1186/s13059-026-04034-w)
Supplement: Supplementary file 3 — Additional file 3. Uncropped scans of all Western blots presented in this study. [file 13059_2026_4034_MOESM3_ESM.pdf]

# Figure 2B

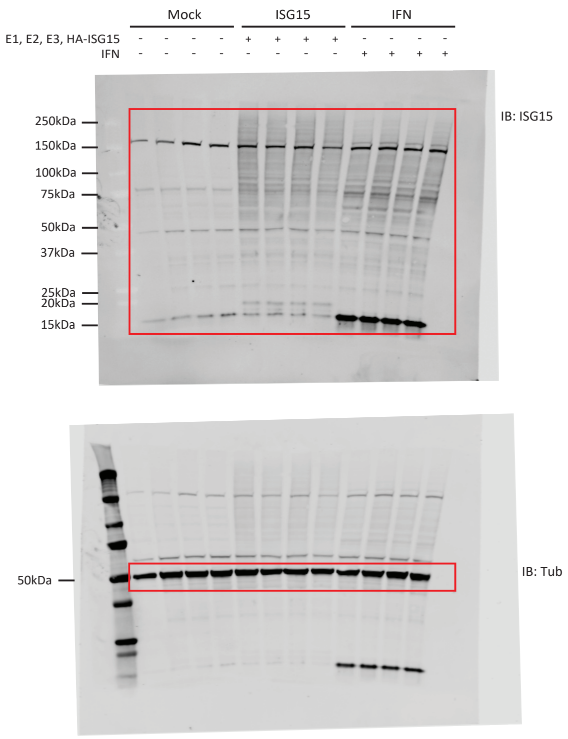
Mock
ISG15
IFN
E1, E2, E3, HA-ISG15 - - - - + + + + - - - -
IFN - - - - - - - - + + + +
250kDa
150kDa
100kDa
75kDa
50kDa
37kDa
25kDa
20kDa
15kDa
IB: ISG15
50kDa
IB: Tub

# Figure 3B

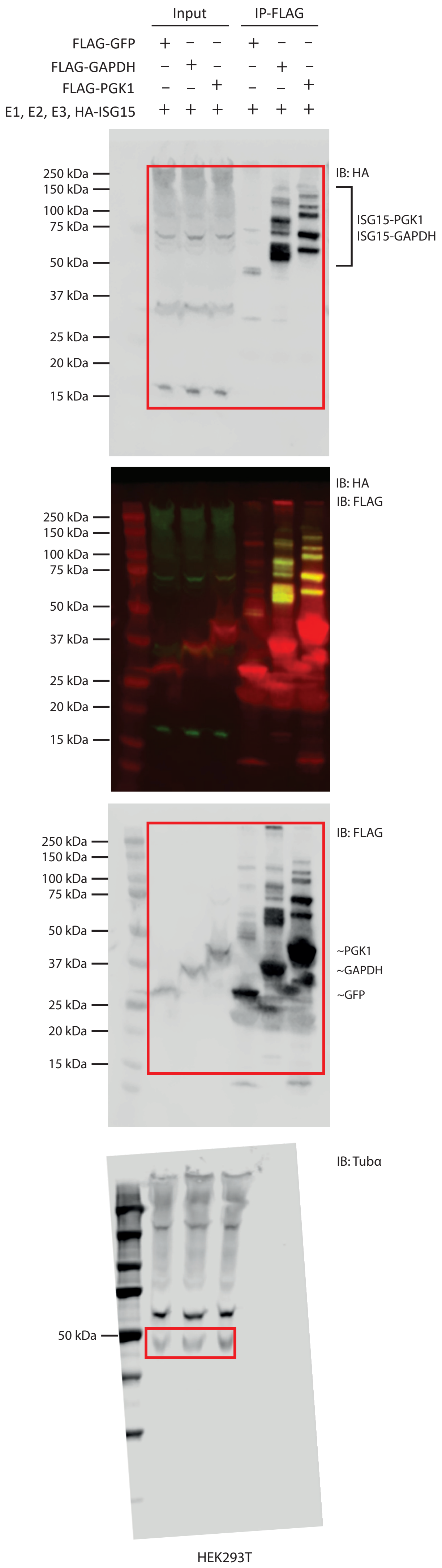
Input
IP-FLAG
FLAG-GFP + – – + – –
FLAG-GAPDH – + – – + –
FLAG-PGK1 – – + – – +
E1, E2, E3, HA-ISG15 + + + + + +
250 kDa
150 kDa
100 kDa
75 kDa
50 kDa
37 kDa
25 kDa
20 kDa
15 kDa
IB: HA
ISG15-PGK1
ISG15-GAPDH
IB: HA
IB: FLAG
IB: FLAG
~PGK1
~GAPDH
~GFP
IB: Tubα
50 kDa
HEK293T

# Figure 4A

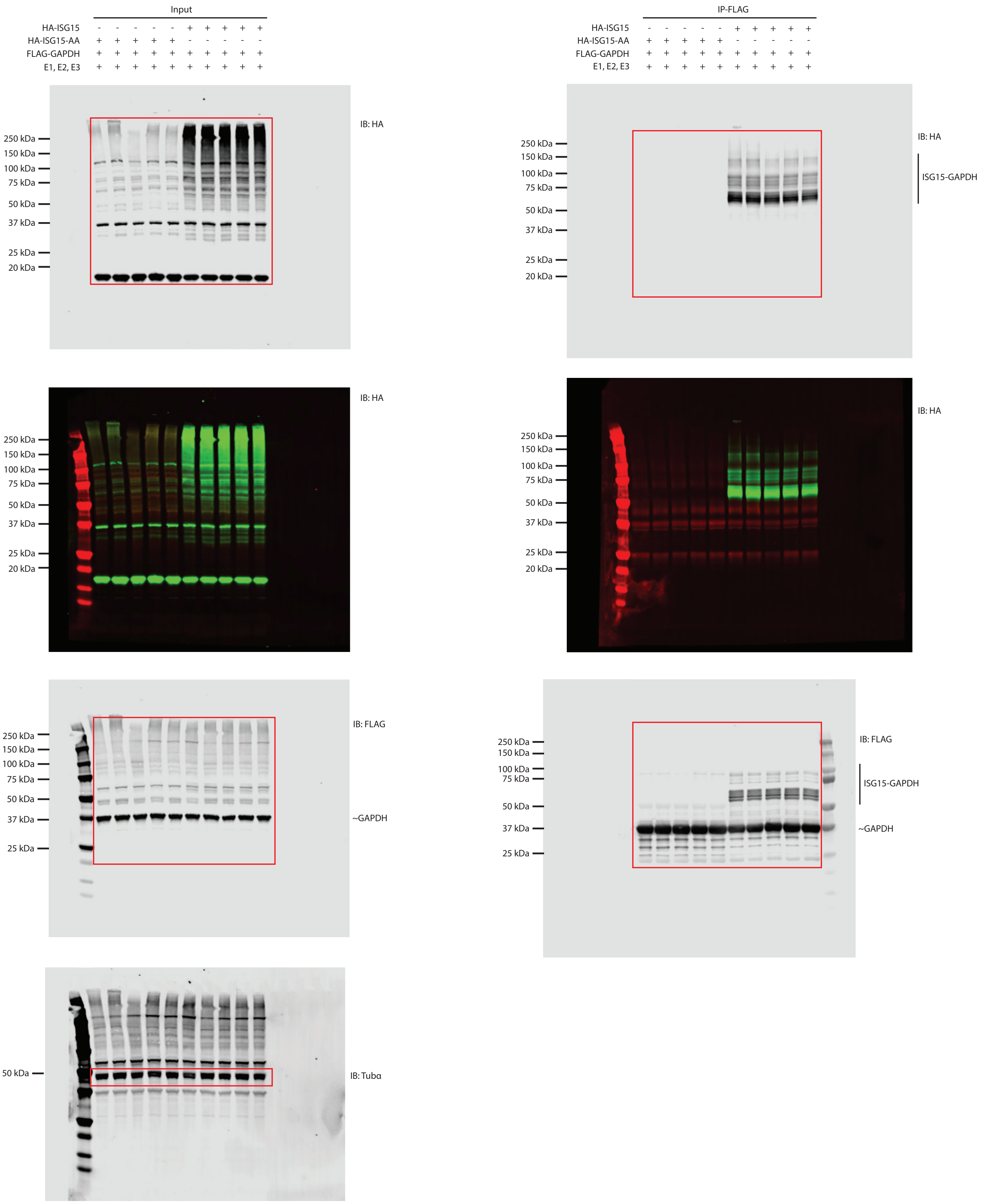

Input
HA-ISG15 - - - - - + + + + +
HA-ISG15-AA + + + + + - - - - -
FLAG-GAPDH + + + + + + + + + +
E1, E2, E3 + + + + + + + + + +
IP-FLAG
HA-ISG15 - - - - - + + + + +
HA-ISG15-AA + + + + + - - - - -
FLAG-GAPDH + + + + + + + + + +
E1, E2, E3 + + + + + + + + + +
250 kDa
150 kDa
100 kDa
75 kDa
50 kDa
37 kDa
25 kDa
20 kDa
IB: HA
ISG15-GAPDH
IB: FLAG
~GAPDH
IB: Tubα

# Figure 4B

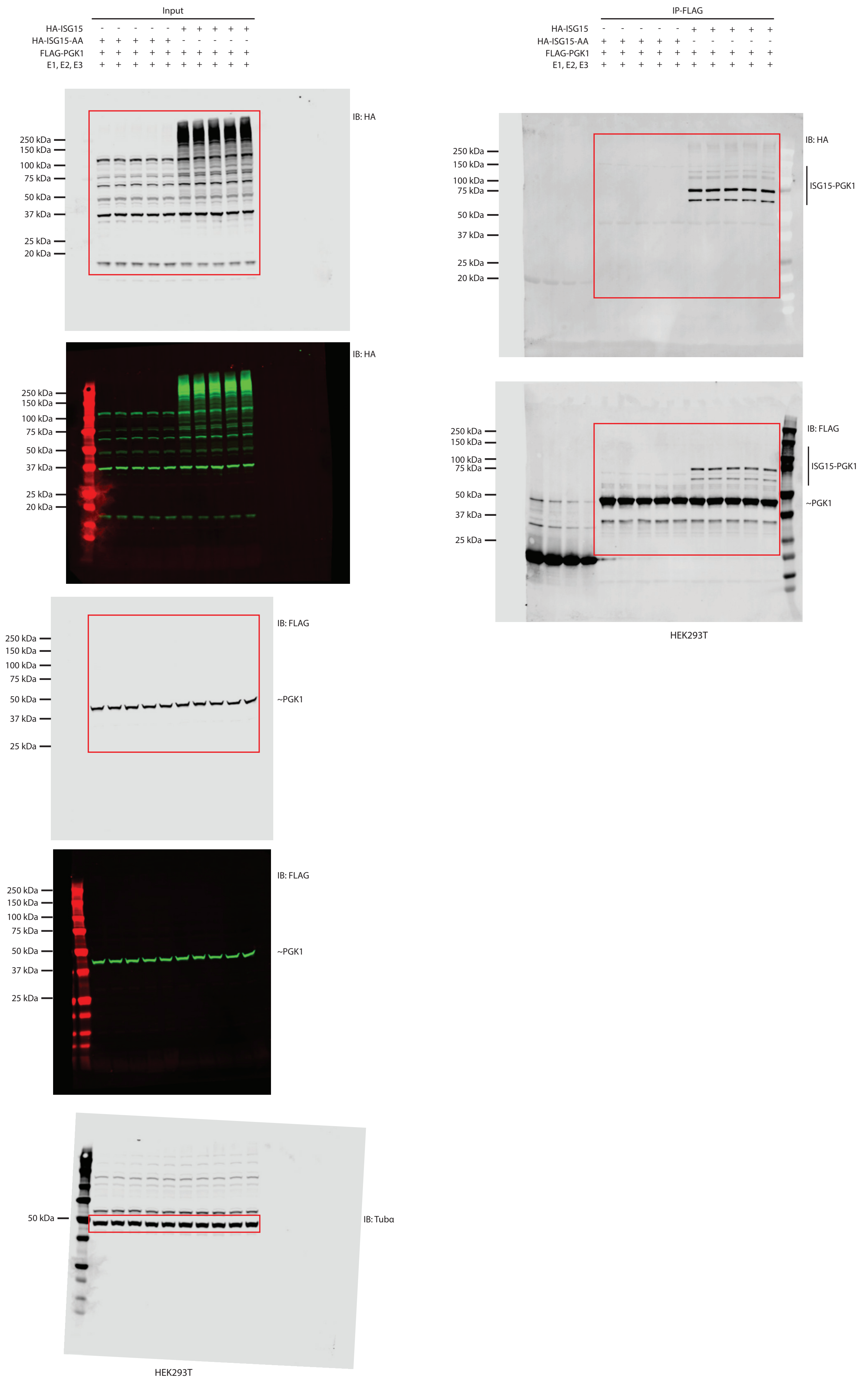
Input
IP-FLAG
HA-ISG15
HA-ISG15-AA
FLAG-PGK1
E1, E2, E3
250 kDa
150 kDa
100 kDa
75 kDa
50 kDa
37 kDa
25 kDa
20 kDa
IB: HA
IB: FLAG
IB: Tuba
ISG15-PGK1
~PGK1
HEK293T

# Figure 5E

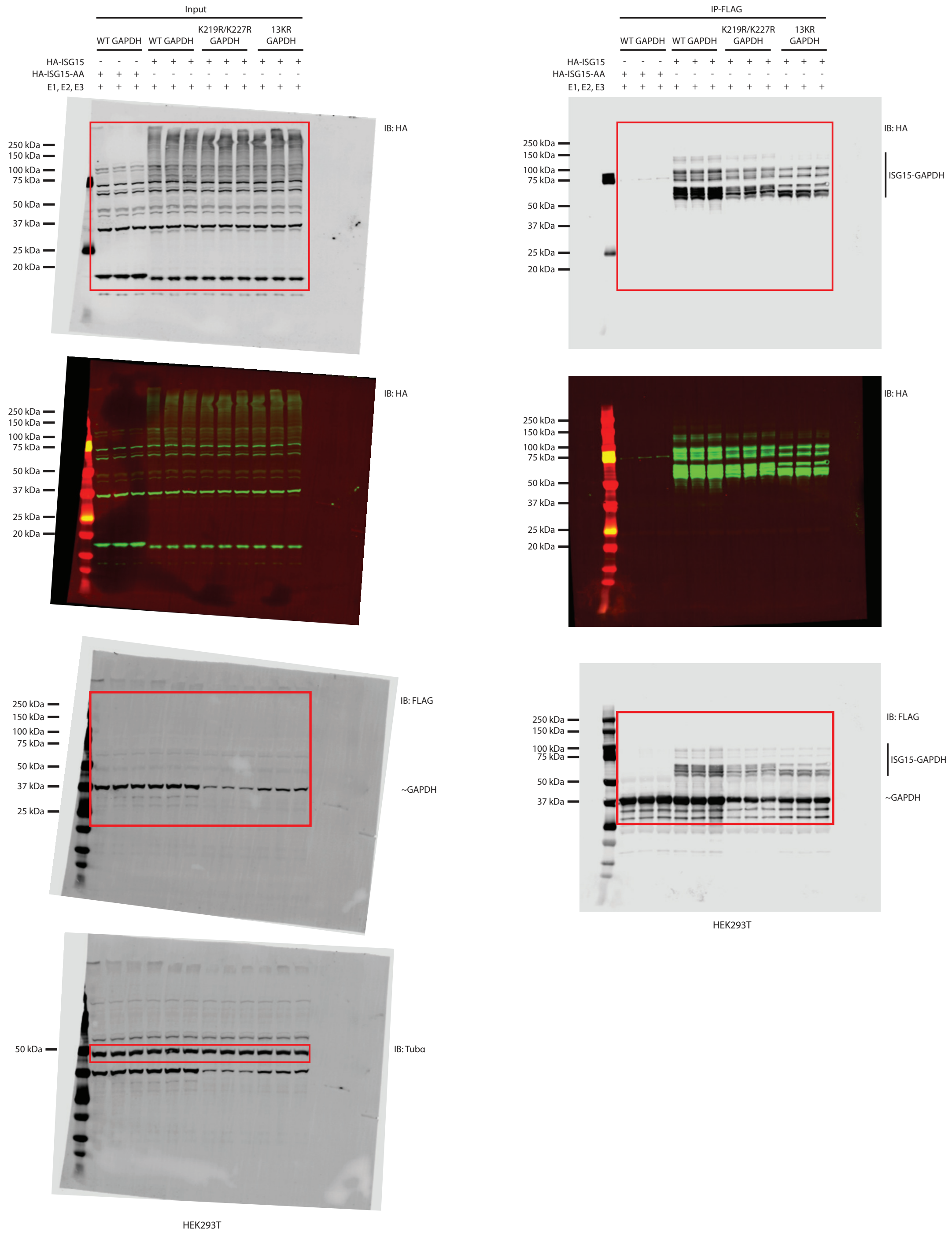
Input
IP-FLAG
WT GAPDH
WT GAPDH
K219R/K227R GAPDH
13KR GAPDH
HA-ISG15
HA-ISG15-AA
E1, E2, E3
250 kDa
150 kDa
100 kDa
75 kDa
50 kDa
37 kDa
25 kDa
20 kDa
IB: HA
ISG15-GAPDH
IB: FLAG
~GAPDH
IB: Tubα
HEK293T

# Figure S1A

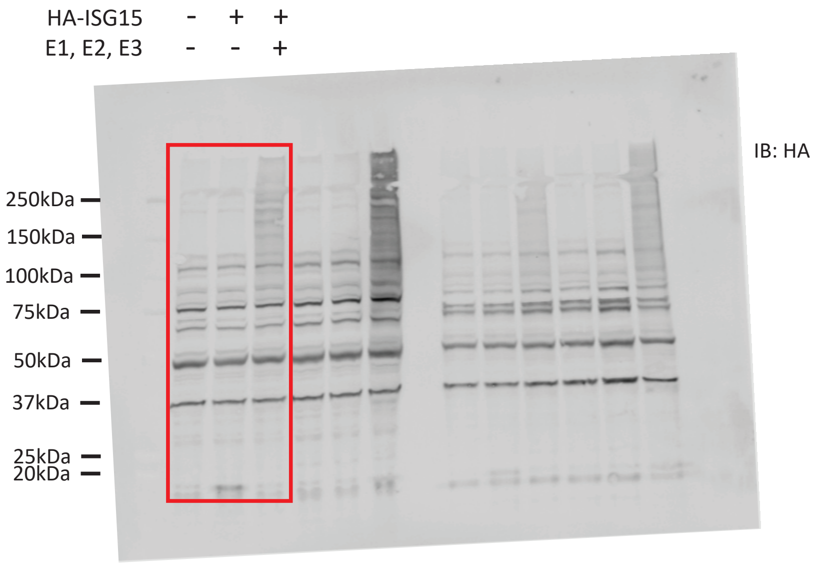

HA-ISG15 - + +
E1, E2, E3 - - +
IB: HA
250kDa
150kDa
100kDa
75kDa
50kDa
37kDa
25kDa
20kDa


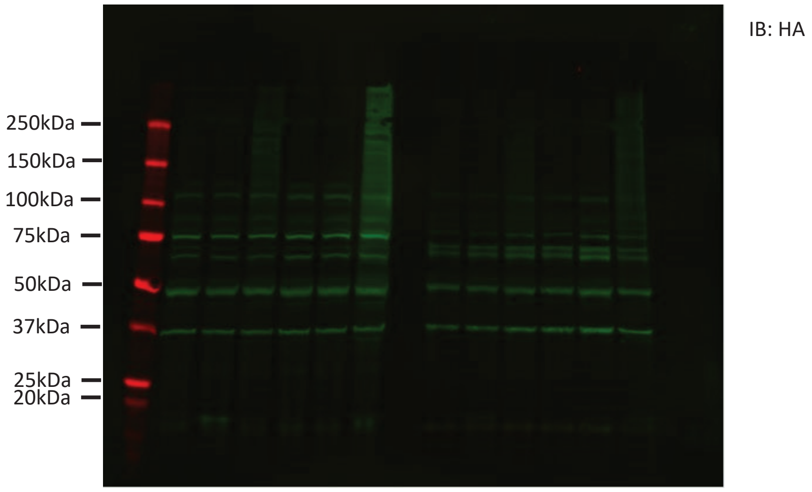

IB: HA
250kDa
150kDa
100kDa
75kDa
50kDa
37kDa
25kDa
20kDa


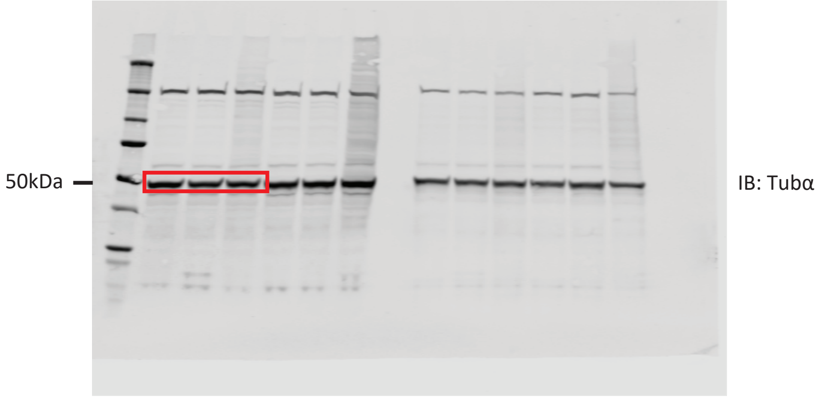

50kDa
IB: Tubα

# Figure S1B

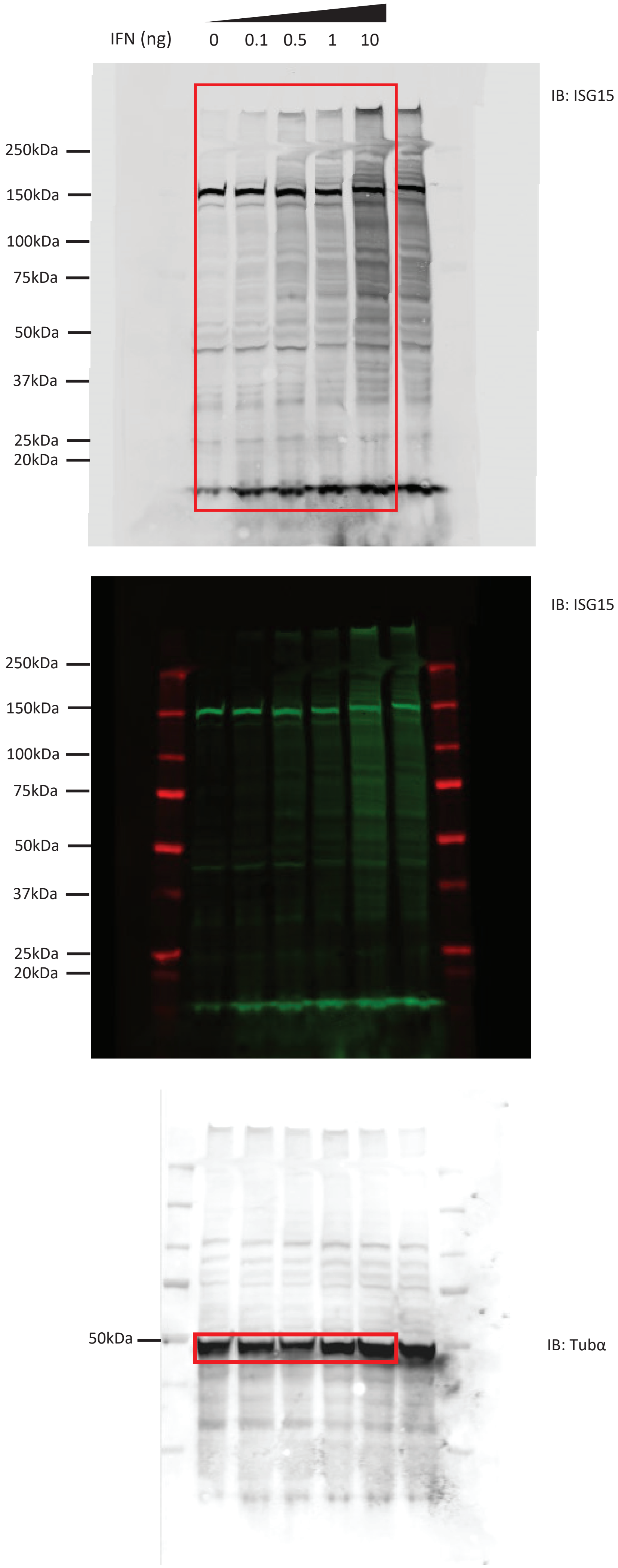
IFN (ng)
0
0.1
0.5
1
10
IB: ISG15
250kDa
150kDa
100kDa
75kDa
50kDa
37kDa
25kDa
20kDa
IB: ISG15
250kDa
150kDa
100kDa
75kDa
50kDa
37kDa
25kDa
20kDa
50kDa
IB: Tubα

# Figure S2

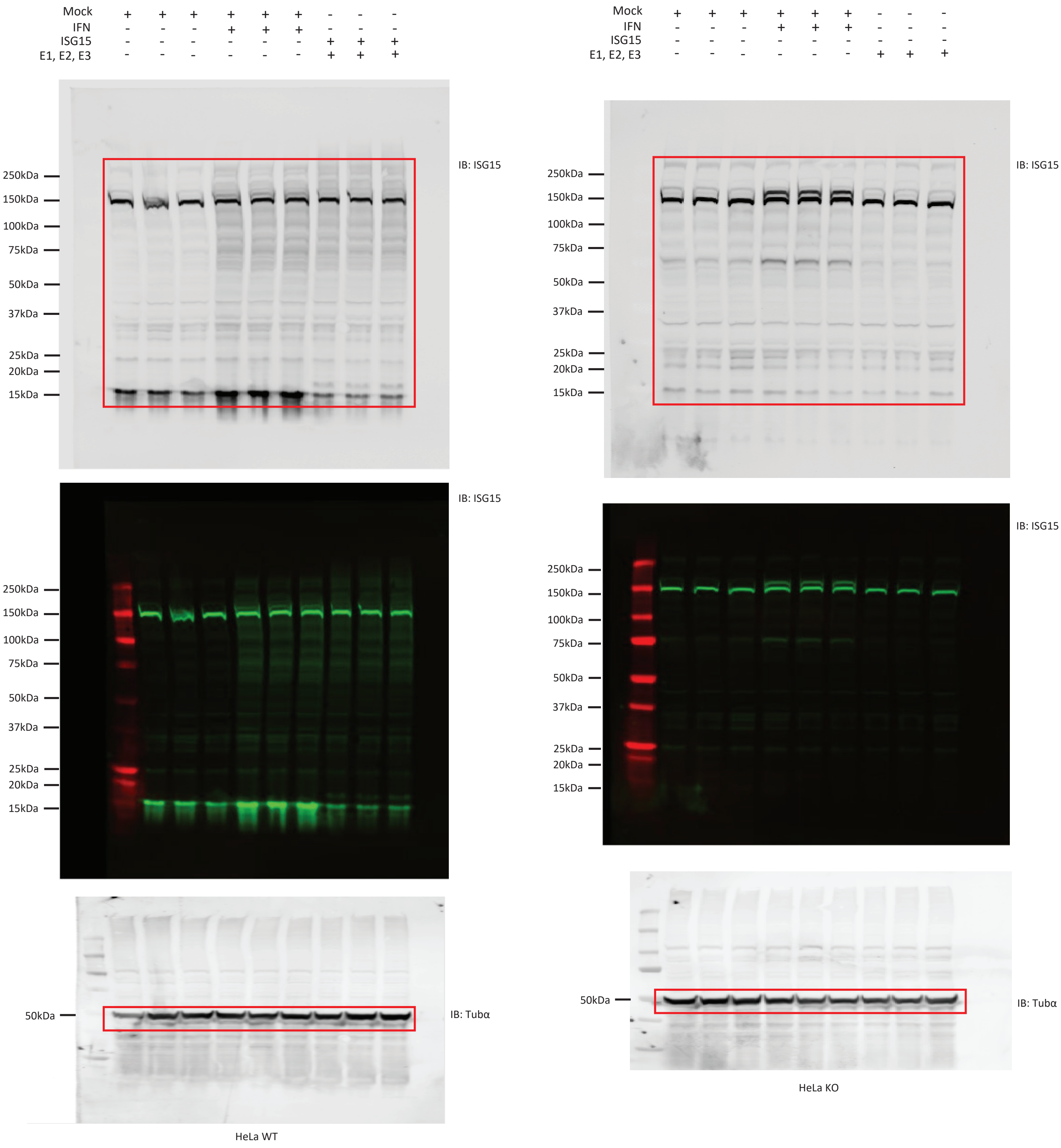
Mock
IFN
ISG15
E1, E2, E3
250kDa
150kDa
100kDa
75kDa
50kDa
37kDa
25kDa
20kDa
15kDa
IB: ISG15
IB: Tubα
HeLa WT
HeLa KO

# Figure S8B

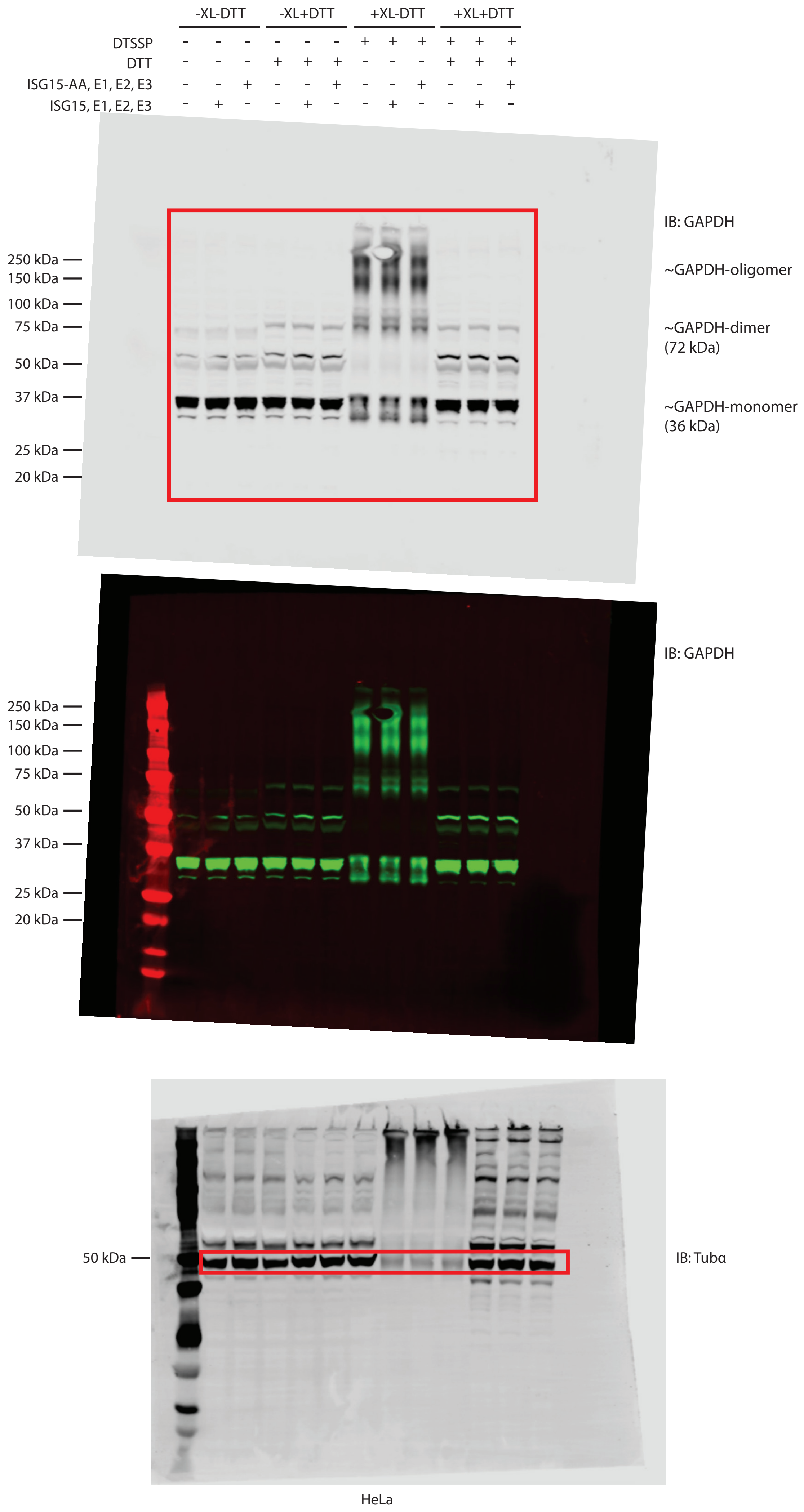
-XL-DTT
-XL+DTT
+XL-DTT
+XL+DTT
DTSSP
DTT
ISG15-AA, E1, E2, E3
ISG15, E1, E2, E3
250 kDa
150 kDa
100 kDa
75 kDa
50 kDa
37 kDa
25 kDa
20 kDa
IB: GAPDH
~GAPDH-oligomer
~GAPDH-dimer (72 kDa)
~GAPDH-monomer (36 kDa)
250 kDa
150 kDa
100 kDa
75 kDa
50 kDa
37 kDa
25 kDa
20 kDa
IB: GAPDH
50 kDa
IB: Tubα
HeLa

# Figure S8C

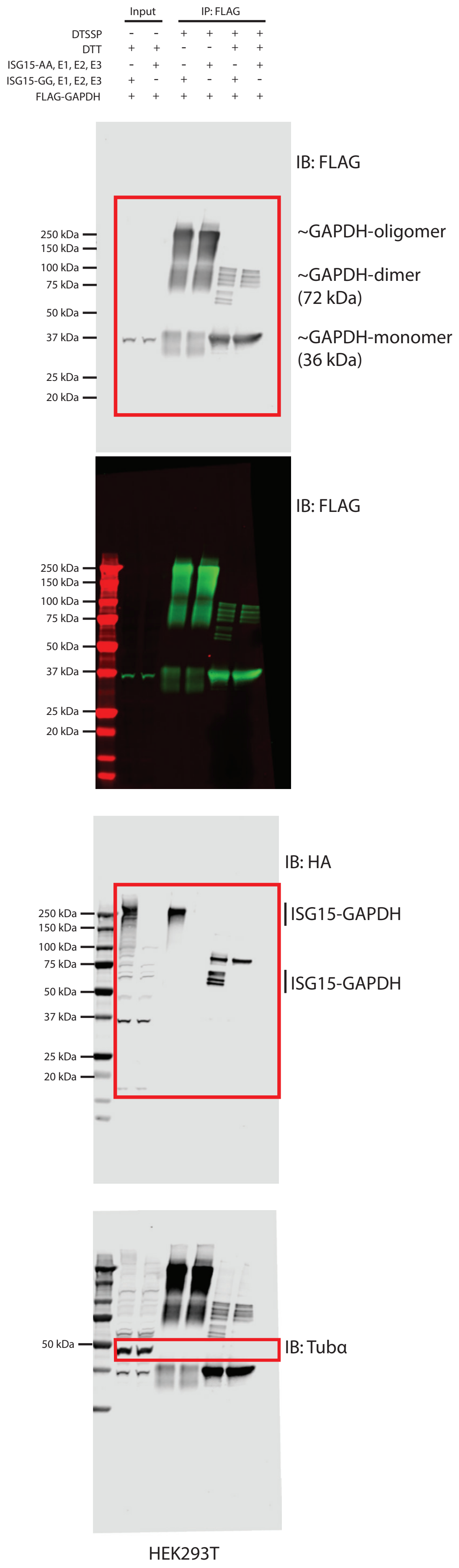

Input
IP: FLAG
DTSSP
DTT
ISG15-AA, E1, E2, E3
ISG15-GG, E1, E2, E3
FLAG-GAPDH
IB: FLAG
250 kDa
150 kDa
100 kDa
75 kDa
50 kDa
37 kDa
25 kDa
20 kDa
~GAPDH-oligomer
~GAPDH-dimer (72 kDa)
~GAPDH-monomer (36 kDa)
IB: FLAG
IB: HA
ISG15-GAPDH
ISG15-GAPDH
IB: Tubα
HEK293T
